# Qing-Yi Decoction in the Treatment of Acute Pancreatitis: An Integrated Approach Based on Chemical Profile, Network Pharmacology, Molecular Docking and Experimental Evaluation

**DOI:** 10.3389/fphar.2021.590994

**Published:** 2021-04-29

**Authors:** Tian-Fu Wei, Liang Zhao, Peng Huang, Feng-Lin Hu, Ju-Ying Jiao, Kai-Lai Xiang, Zhi-Zhou Wang, Jia-Lin Qu, Dong Shang

**Affiliations:** ^1^ Laboratory of Integrative Medicine, The First Affiliated Hospital of Dalian Medical University, Dalian, China; ^2^ Institute (College) of Integrative Medicine, Dalian Medical University, Dalian, China; ^3^ Department of General Surgery, Pancreatic-Biliary Center, The First Affiliated Hospital of Dalian Medical University, Dalian, China

**Keywords:** Qing-Yi decoction, acute pancreatitis, chemical profile, network pharmacology, molecular docking, p65, ERK1/2, c-fos

## Abstract

**Background:** Qing-Yi Decoction (QYD) is a classic precompounded prescription with satisfactory clinical efficacy on acute pancreatitis (AP). However, the chemical profile and overall molecular mechanism of QYD in treating AP have not been clarified.

**Methods:** In the present study, a rapid, simple, sensitive and reliable ultra-performance liquid chromatography coupled with quadrupole time-of-flight mass spectrometry (UHPLC-QTOF-MS)-based chemical profile was first established. An integration strategy of network pharmacology analysis and molecular docking based identified ingredients was further performed to screen out the potential targets and pathways involved in the treatment of QYD on AP. Finally, SD rats with acute pancreatitis were constructed to verify the predicted results through a western blot experiment.

**Results:** A total of 110 compounds, including flavonoids, phenolic acids, alkaloids, monoterpenes, iridoids, triterpenes, phenylethanoid glycosides, anthraquinones and other miscellaneous compounds were identified, respectively. Eleven important components, 47 key targets and 15 related pathways based on network pharmacology analysis were obtained. Molecular docking simulation indicated that ERK1/2, c-Fos and p65 might play an essential role in QYD against AP. Finally, the western blot experiments showed that QYD could up-regulate the expression level of ERK1/2 and c-Fos, while down-regulate the expression level of p65.

**Conclusion:** This study predicted and validated that QYD may treat AP by inhibiting inflammation and promoting apoptosis, which provides directions for further experimental studies.

## Introduction

Acute pancreatitis (AP) is a common clinical disease caused by a variety of factors, including cholelithiasis, excessive drinking, obesity, smoking, etc (Sadr-Azodi et al., 2012; Yadav and Lowenfels, 2013; Lankisch et al., 2015; Forsmark et al., 2016), which is manifested by acute epigastric pain, nausea, vomiting and fever (Lankisch et al., 2015). The overall incidence rate of AP is 13–45 per 100,000 persons and is increasing year by year (Yadav and Lowenfels, 2013; Lankisch et al., 2015).

At present, the western medicine treatment of AP is mainly focused on spasmolysis, pain relief, inhibition of pancreatin secretion and fluid resuscitation. In severe cases, surgical treatment will be used (Lankisch et al., 2015; Brunschot et al., 2018). However, the therapeutic effect of these schemes is limited because they can only control the symptoms without an ultimate cure. Furthermore, there is no specific drug available yet. Therefore, it is imperative to develop safer and more effective drugs to treat AP. Traditional Chinese medicine (TCM), which has a history of clinical application for thousands of years in China, has been gradually accepted in the treatment of AP in view of its characteristics of holism concept and pattern differentiation (Wang and Zhang, 2017) as well as advantages of less side effects and systemic regulation.

Qing-Yi Decoction (QYD) is a classic precompounded prescription that consists of eight herbs, namely, *Rheum officinale* Baill. (da-huang in Chinese), *Bupleurum chinense* DC. (chai-hu in Chinese), *Scutellaria baicalensis* Georgi (huang-qin in Chinese), *Paeonia lactiflora* Pall. (bai-shao in Chinese), *Aucklandia costus* Falc. (mu-xiang in Chinese), *Corydalis yanhusuo* (Y. H. Chou and Chun C. Hsu) W. T. Wang ex Z. Y. Su and C. Y. Wu (yan-hu-suo in Chinese), *Gardenia jasminoides* J. Ellis (zhi-zi in Chinese) and *Natrii* Sulfas (mang-xiao in Chinese). Previous studies reported that it could treat AP by reducing the production of various inflammatory mediators, blocking inflammatory signaling pathways, and improving intestinal mucosal barrier and motility effectively (Zhang et al., 2015). In clinical practice, QYD is more widely used than similar prescriptions such as Da-Cheng-Qi Decoction because of its advantages of targeting symptomatic infection by heat-clearing, detoxifying and removing stasis by purgation. (Qu et al., 2007; Chen et al., 2015). However, there are some shortages in the current research on QYD. For one thing, the pharmacodynamic material basis and quality evaluation system related to drug efficacy of QYD have not been established. For another, the current reports are mostly limited to a single target, which is difficult to reflect the “holism” characteristics of QYD as a compound preparation with multiple constituents, multiple targets and multiple pathways, and lacks convincing power. The above problems hindered the development and application of QYD. Therefore, it is necessary to characterize and reveal its chemical constituents and overall mechanism systematically.

Network pharmacology was proposed by Andrew Hopkins in 2007. Its core idea is to optimize treatment strategies depends on a biological network formed by disease features, bioactive agents and drug targets that connect to each other (Hopkins, 2007). It has been introduced to evaluate the constituents and action mechanisms of TCM in view of its systematic and holistic coincides (Li et al., 2011; Li and Zhang, 2013; Cai et al., 2018a). Molecular docking is a technology of drug design and screening based on computer data simulation through the interaction and affinity between receptor macromolecules and drug micromolecules (Chen et al., 2014; Lionta et al., 2014; Saikia and Bordoloi, 2019; Reddyrajula and Dalimba, 2020). At present, their combined use has been successfully applied in the research of Traditional Chinese medicine and their compound preparation (Liang et al., 2019; Wang et al., 2019).

**FIGURE 1:**
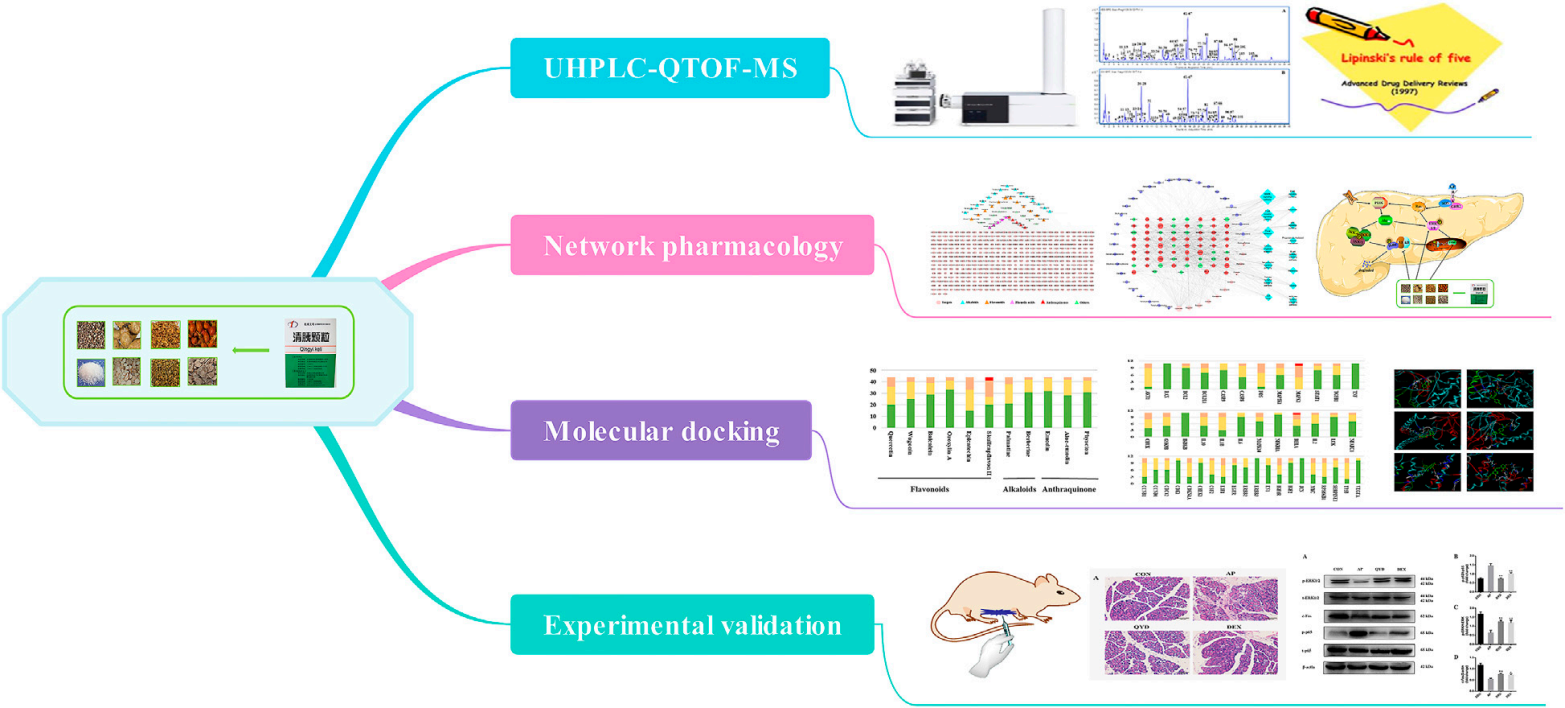
The schematic diagram of the present study.

In this study, we systematically expounded the possible targets and related pathways of QYD in treating AP by integrating the ultra-performance liquid chromatography coupled with quadrupole time-of-flight mass spectrometry (UHPLC-QTOF-MS), network pharmacology, molecular docking analysis and experimental evaluation using molecular biology. The schematic diagram of this study was shown in Figure 1.

## Materials and Methods

### Chemicals, Reagents and Materials

Methanol and acetonitrile of HPLC grade were purchased from Merck Company Inc. (Darmstadt, Germany). The chemical of formic acid was MS grade and purchased from Fisher Scientific Company Inc. (Fairlawn, NJ). All other reagents were of analytical grade and supplied by Tianjin Concord Technology Co., Ltd. (Tianjin, China). Ultra-pure water (18.2 MΩ) was prepared daily with a Milli‐Q water purification system (Millipore, Milford, MA, United States).

Gallic acid (**3**), catechin (**18**), chlorogenic acid (**19**) (+)-epicatechin (**28**), scutellarin (**36**), tetrahydropalmatine (**47**), jatrorrhizine (**48**), baicalin (**62**), palmatine (**72**), baicalein (**88**), aloe-emodin (**93**), rhein (**94**), saikosaponin a (**96**), wogonin (**97**), emodin (**104**), chrysophanol (**107**), and physcion (**108**) were obtained from the National Institutes for Food and Drug Control (Beijing, China). Syringin (**22**), albiflorin (**29**), paeoniflorin (**31**), rutin (**35**) and benzoylpaeoniflorin (**85**) were purchased from Chengdu Pufei De Biotech (Chengdu, Sichuan, China). The purity of each reference standard was above 98%.

All the eight herbs of QYD, including *Rheum officinale* Baill. (batch No. 160702), *Bupleurum chinense* DC. (batch No. 170509), *Scutellaria baicalensis* Georgi (batch No. 170505), *Paeonia lactiflora* Pall. (batch No. 170308), *Aucklandia costus* Falc. (batch No. 170503), *Corydalis yanhusuo* (Y. H. Chou and Chun C. Hsu) W. T. Wang ex Z. Y. Su and C. Y. Wu (batch No. 170307), *Gardenia jasminoides* J. Ellis (batch No. 170305) and *Natrii* Sulfas (batch No. 170609) were purchased from the First Affiliated Hospital of Dalian Medical University (Dalian, Liaoning Province, China), and authenticated by Professor Aijing Leng (Department of Chinese medicine, The First Affiliated Hospital of Dalian Medical University). Their voucher specimens were deposited at the author’s laboratory.

### Preparation of Qing-Yi Decoction Extract and Standard Solution

Raw herbs for the formula (containing *Bupleurum chinense* DC., 15 g; *Scutellaria baicalensis* Georgi, 12 g; *Paeonia lactiflora* Pall., 15 g; *Aucklandia costus* Falc, 15 g; *Corydalis yanhusuo* (Y. H. Chou and Chun C. Hsu) W. T. Wang ex Z. Y. Su and C. Y. Wu, 15 g; and *Gardenia jasminoides* J. Ellis, 15 g) was soaked and extracted by boiling with 10-fold mass of water (870 ml) for 1 h. After filtered with six-layer absorbent gauze, the residue was re-extracted with 8-fold mass of water in the same way for 0.5 h. Then *Rheum officinale* Baill. (20 g) was added into the extract and boiled for additional 0.5 h. After being filtered with six-layer absorbent gauze and added by *Natrii* Sulfas (10 g) when solution was hot, the two filtrates were combined and concentrated under vacuum to 120 ml (equal to 1 g crude herb/ml), and finally the concentrate was subjected to freeze-dry. The extract was obtained with a yield of 29.5% and kept in the desiccator before use.

A 1.0 g of the freeze-dried powder was accurately weighted and extracted with 50 ml of methanol/water (1:1, v/v) for 30 min under ultrasound. The extract solution was centrifuged at 13,000 rpm for 10 min at 4°C and the supernatant was filtered through a 0.22 μm membrane filters. Finally, 1.0 μl of filtrate was injected into UHPLC-QTOF-MS for analysis.

### Ultra-Performance Liquid Chromatography Coupled With Quadrupole Time-of-Flight Mass Spectrometry Condition

Chromatographic separation was carried out on an Agilent 1,290 Infinity LC system (Agilent, United States) using an Agilent Zorbax Eclipse Plus C18 column (100 × 2.1 mm i.d., 3.5 μm) at 40°C with a flow rate of 0.3 ml/min. The mobile phase consisted of water containing 0.1% formic acid (solvent system A) and acetonitrile (solvent system B) were performed with gradient elution program as follows: 0–5 min, 3–10% B; 5–13 min, 10–18% B; 13–20 min, 18–25% B; 20–35 min, 25–70% B; 35–40 min, 70–99% B; 40–42 min, 99–3% B; 42–45 min, 3% B. 2 μl of sample solution was injected for analysis.

Mass detection was performed using an Agilent 6530b Accurate-Mass Quadrupole Time-of-Flight (Q-TOF) mass spectrometer (Agilent Corp., United States) equipped with a Dual AJS ESI source operating in both positive and negative mode with the following operating parameters: drying gas (N_2_) flow rate, 10.0 L/min; drying gas (N_2_) temperature, 350°C; nebulizer, 35 psig; sheath gas (N_2_) temperature, 400°C; fragmentor voltage, 120 V; skimmer voltage, 65 V; Octopole RF, 750 V. The capillary voltage was set at 4 kV or −3.5 kV under positive or negative mode, respectively. The nozzle voltage was set at +500 V or −1000 V, respectively; The quasi-molecular ions [M − H]^−^ and [M + H]^+^ were selected as precursor ions and subjected to target-MS/MS analyses with different collision energies ranging from 10 to 60 V to acquire sufficient product ions. MS spectra were recorded over the m/z range of 50–1,100. All data was processed by MassHunter workstation software version B.06.00 (Agilent Technologies, Germany).

### Network Pharmacology Analysis

#### Establishment of Component Target Database for Qing-Yi Decoction

Briefly, the constituents contained in QYD identified by UHPLC-QTOF-MS were input into the Molispiration Smiles database (https://www.molinspiration.com/cgi-bin/properties), and the active constituents were screened out through the Lipinski’s rule of five (Lipinski et al., 2001). Then, the Canonical SMILES structure formats of these constituents were obtained from Pubchem database (https://www.ncbi.nlm.nih.gov/pubmed/), and the component targets were found out by inputting them into Swiss Target Prediction Database (http://www.swisstargetprediction.ch/). To obtain more target information, the names of the ingredients were also input into Traditional Chinese Medicine Systems Pharmacy Database and Analysis Platform (TCMSP) (http://LSP.nwu.edu.cn/tcmsp.php). Finally, the UniProt Knowledgebase (http://www.uniprot.org/) was used to convert the protein names into official gene symbols (*Homo sapiens*).

#### Establishment of Therapeutic Target Database for Acute Pancreatitis

The information of therapeutic target was searched by using “acute pancreatitis” as the keyword. After integration of search-derived target data and elimination of the repeated genes, the therapeutic targets database can be obtained. The databases that used in this study were shown below.

1) OMIM database (http://omim.org/)
2) GeneCards database (https://www.genecards.org/)
3) DisGeNET database (http://www.disgenet.org/web/DisGeNET/menu)
4) GAD database (https://geneticassociationdb.nih.gov/)

#### The Protein-Protein Interactions Network Analysis

To clarify the relationship between intersection targets, an intersection targets protein-protein interactions (PPI) network of acute pancreatitis and QYD component was constructed and analyzed using STRING database (https://string-db.org/). Species were defined as Homo sapiens. PPIs with a confidence score greater than 0.95 were selected and topology analyses were carried out to ensure the accuracy of the results. Finally, targets with a degree value greater than twice the median were chosen for subsequent network construction and analysis (Lu et al., 2017; Li et al., 2018a).

#### Enrichment Analysis

In order to clarify the signaling pathways related to key targets, Kyoto Gene and Genome Encyclopedia (KEGG) enrichments analysis was performed based on Database for Annotation, Visualization and Integrated Discovery (DAVID, https://david.ncifcrf.gov/home.jsp, ver. 6.8) (Huang et al., 2009).

#### Network Construction and Analysis

To visualize the research data, we process the data by employing the network visualization software Cytoscape 3.2.1 (Demchak et al., 2014), which supplies a method for data integration, analysis, and visualization for complex network analysis. The two networks were constructed as follows: 1) constituents-putative targets network of QYD; 2) active constituents-intersection target PPIs-pathways network. In these network diagrams, constituents, targets, and pathways were all represented by nodes, while the edges indicate the interactions between different nodes. The “degree” of a node was determined by the number of connected edges.

### Molecular Docking

The Surflex-docking module in SYBYL-X 2.0 (Boström, 2001) was performed to evaluate the binding ability between the screened core constituents and targets. First of all, the 2D structure of the compound was obtained from the PubChem database (https://www.ncbi.nlm.nih.gov/pubmed/) in SDF format and then converted into the 3D structure in mol2 format by SYBYL-X 2.0 software for the preparation of micromolecule ligand compounds. Next, the protein crystal structure of core targets was obtained from the RCSB Protein Data Bank database (http://www.rcsb.org) after searching PDB ID in the uniport database (http://www.uniprot.org/). Finally, a series of treatments include removing water molecules and original ligands, adding hydrogen, repairing amino acids and forming binding pockets were performed to complete the preparation of macromolecular receptor target proteins. “Total Score” given by software was used as the indicator for docking results. The higher the score, the stronger the binding effect.

### Experiments Evaluation

#### Animals and Drug Treatments

Twenty four male Sprague-Dawley (SD) rats (240 ± 10 g body weight about 6–8 weeks-old) purchased from the Laboratory Animal Center of Dalian Medical University (License number: SCXK (Liao) 2018-0003) were maintained in a breeding room under controlled temperature (22 ± 2°C), humidity (55 ± 5%) and lighting (12 h light-dark cycles) conditions. To be acclimatized, rats were allowed food and water *ad libitum* for one week before the experiments. All experimental protocol was approved by the Ethics Review Committee for Animal Experimentation of Dalian Medical University.

SD rats were randomly divided into four groups including the control (CON) group, acute pancreatitis (AP) group, Qing-Yi Decoction-treated (QYD) group and dexamethasone-treated (DEX) group (*n* = 6 in each group). AP model was induced by retrograded injection of 3.5% sodium taurocholate (STC) into the pancreatic duct according to the previous report (Huang et al., 2017). For the CON group, only the traction of the duodenum was conducted, and injection of the drug wasn’t given. QYD extract was orally administrated to rats at a dosage of 10 g crude drug/kg/day when awakening after modeling. DEX that dissolved with normal saline by ultrasound was used as a positive drug at a dose of 10 mg/kg by intravenous tail injection. QYD and DEX were given repeated 12 h later. Rats in the control and model group were given physiological saline, which had the same volume as QYD and DEX.

After modeling for 24 h, rat serum was collected by centrifugation at 3,000 rpm for 10 min at 4°C. Meanwhile, part of pancreatic tissues was collected and fixed in 4% paraformaldehyde solution for histopathological staining. The remaining ones were immediately frozen and stored at −80°C for western blot analysis.

#### Histopathological Examination and Biochemical Analysis

Pancreatic was observed under an optical microscope (Olympus, Japan) with a magnification of 200 times after embedded in paraffin and stained with hematoxylin and eosin. Finally, the previous scoring system was used to evaluate the damage degree of pancreatic tissue with edema, inflammation and vacuolation as indexes (Rongione et al., 1997). Also, the serum of rats was diluted 100 times, and then the amylase level was determined according to the instructions of the kit (Jiancheng, Nanjing, China).

#### Western Blot Analysis

Total protein was extracted from pancreas tissues with cold lysis buffer and PMSF protease inhibitor. Next, the protein concentration was determined using the BCA protein assay kit. The protein samples were fractionated by SDS-PAGE (8–10%) and then transferred to PVDF membranes. The membranes were blocked with 5% non-fat milk for 1.5 h and incubated overnight at 4°C with the following primary antibodies: p65, p-p65, ERK1/2, p-ERK1/2, c-Fos, β-actin (1:1,000 dilution). The membranes were incubated 1 h at room temperature with secondary antibodies and protein expression was probed with an ECL method and placed in a Tanon-5200 Imaging System (Tanon, Shanghai, China).

#### Statistical Analysis

Statistical analysis was carried out with SPSS 20.0 software. All the data in the experiment are expressed as mean ± standard deviation. F-test was used to analyze the variance homogeneity of the data. Then one-way ANOVA’s “LSD test” or “Games-Howell test” was used to compare the differences between the two groups according to the results. Values of *p* < 0.05 were considered statistically significant.

## Results

### Chemical Profile of Qing-Yi Decoction by Ultra-Performance Liquid Chromatography Coupled With Quadrupole Time-of-Flight Mass Spectrometry

**FIGURE 2:**
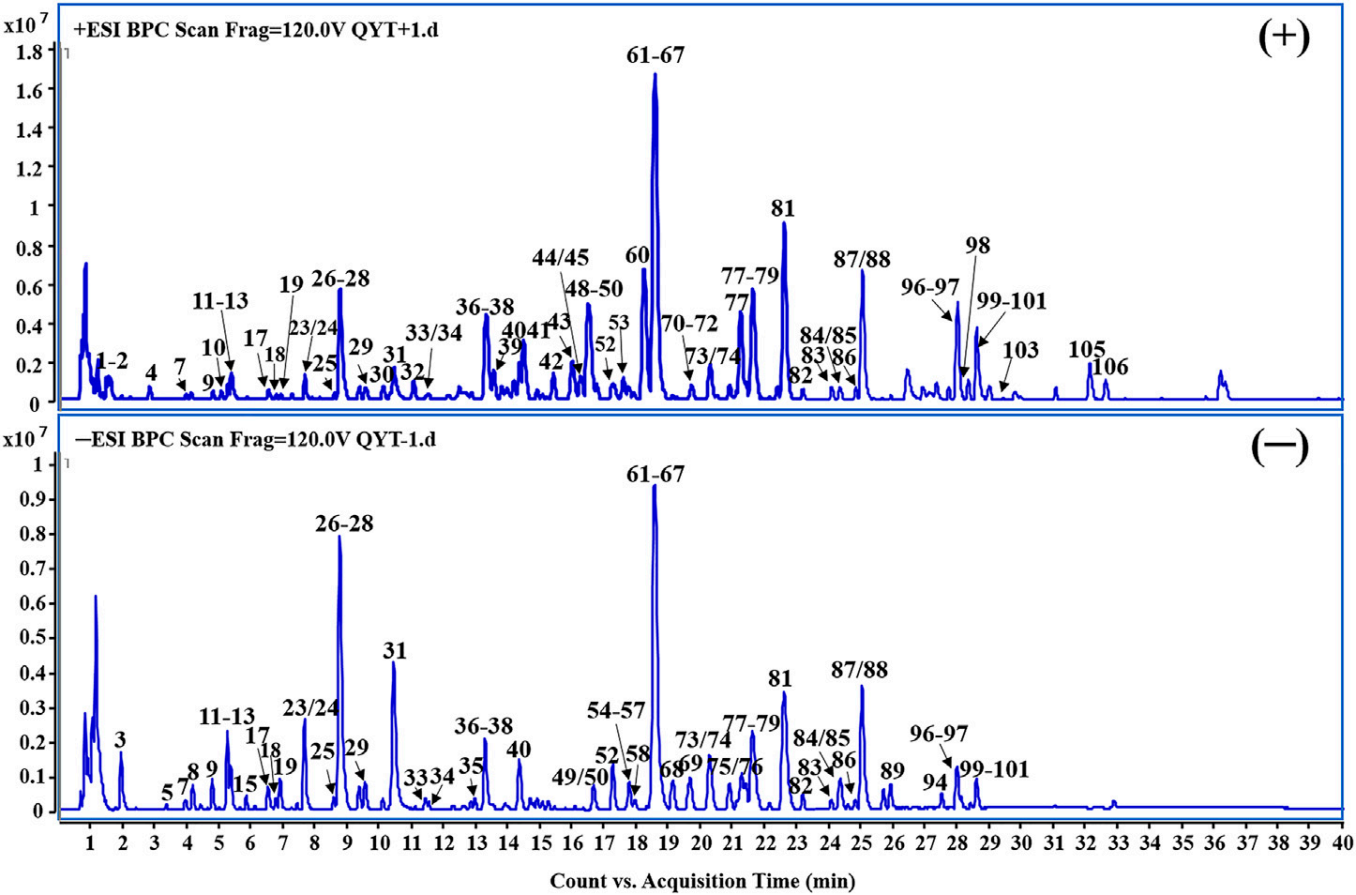
Representative base peak chromatogram (BPC) of QYD in the positive and negative ions mode, respectively.

Under the optimized conditions, a total of 110 constituents including 28 flavonoids, 16 phenolic acids, 15 alkaloids, 15 monoterpenes, 11 iridoids, eight triterpenes, five phenylethanoid glycosides, five anthraquinones and seven miscellaneous compounds that originate from seven herbs were identified or tentatively characterized according to the in-house constituents database established for QYD (Figure 2, Supplementary Table S1). While the typical constituents of *Natrii* Sulfas (Na_2_SO_4_·10H_2_O) were not detected.

Among them, 22 compounds (compounds **3**, **18**, **19**, **22**, **28**, **29**, **31**, **35**, **36**, **47**, **48**, **62**, **72**, **85**, **88**, **93**, **94**, **96**, **97**, **104**, **107** and **108**) were identified as gallic acid, catechin, chlorogenic acid, syringing (+)-epicatechin, albiflorin, paeoniflorin, rutin, scutellarin, tetrahydropalmatine, jatrorrhizine, baicalin, palmatine, benzoylpaeoniflorin, baicalein, aloe-emodin, rhein, saikosaponin a, wogonin, emodin, chrysophanol and physcion by comparing the retention time, quasi-molecular ions with authentic standards, respectively. While the others were tentatively deduced based on their high-accurate quasi-molecular ion such as [M − H]^−^ [M + HCOO]^−^ [M + Cl]^−^ [M]^+^ [M + H]^+^ and [M + Na]^+^ with those of the known published compounds recorded in the in-house library. Information regarding the 110 constituents, such as t_R_ (min), identification, formula, negative ion (m/z), positive ion (m/z) and botanical source, is offered in Supplementary Table S1.

### Network Pharmacology Analysis

#### Qing-Yi Decoction Component Targets Network and Acute Pancreatitis Related Therapeutic Targets

**FIGURE 3:**
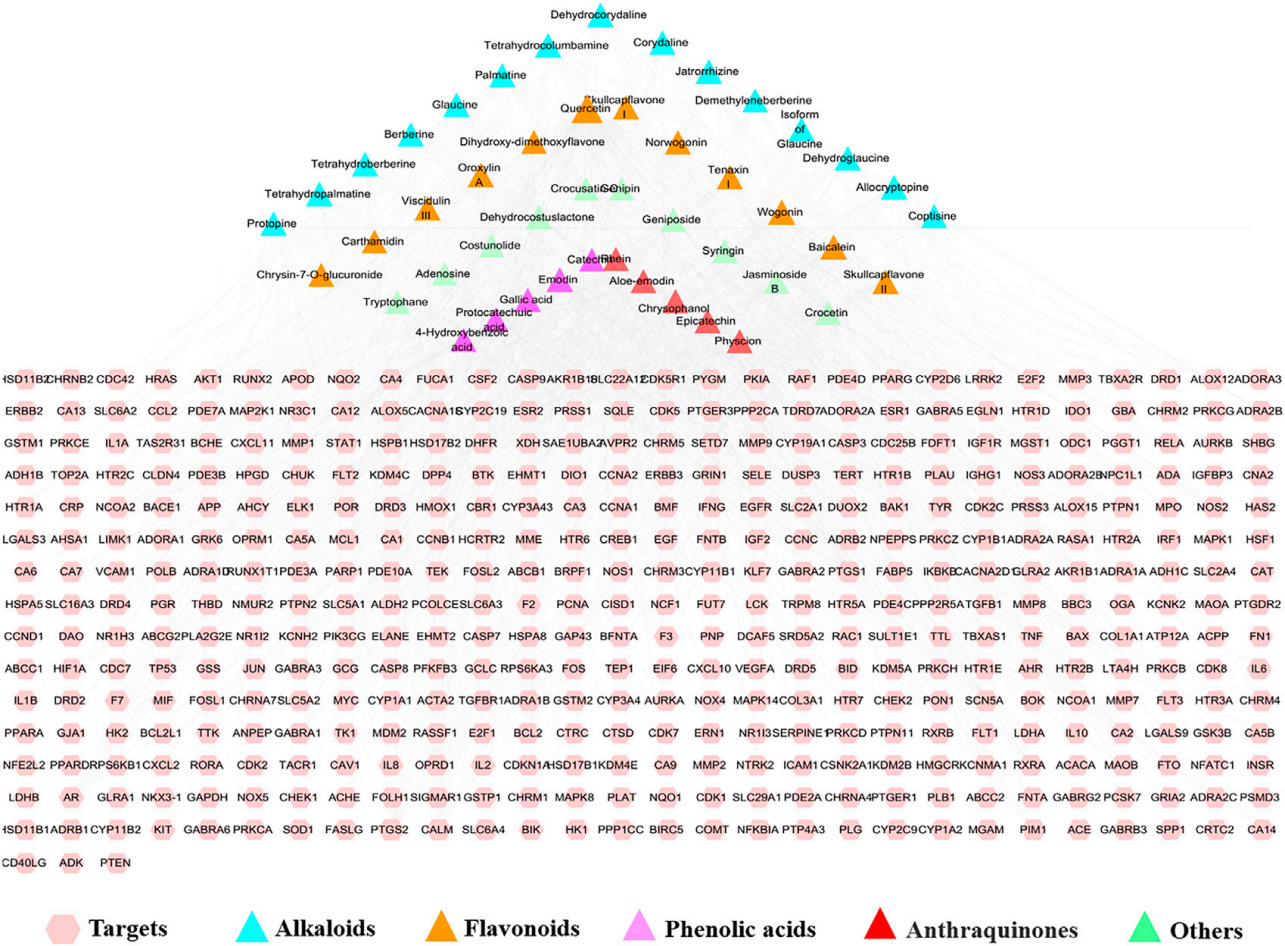
Compound-target network of QYD (Pink circle represents target; Triangle represents compound in QYD. It’s a positive proportional relationship that between the node size and the degree).

Based on the chemical profile of QYD characterized by UHPLC/QTOF-MS, 541 targets associated with the 110 constituents were predicted as potential targets of QYD (Supplementary Table S2, Supplementary Figure S1). In order to focus on more important information, a total of 47 active constituents were screened out according to the Lipinski's rule of five. Correspondingly, the potential targets of the constituents were reduced to 423. The active constituents-potential targets network of QYD for AP was constructed and shown in Figure 3. Similarly, 6593AP-related targets obtained from OMIM, GeneCards, DisGeNET and GAD databases were collected after searching, integrating and de-duplicating steps. (Supplementary Table S3).

#### Acquisition of Candidate Targets and Core Constituents for Qing-Yi Decoction Against Acute Pancreatitis

For the acquisition of the core targets for QYD against AP, the constituents-targets and disease-targets databases were intersected by Cytoscape 3.2.1 software, and 350 intersection targets were obtained. The PPI network of intersection targets was analyzed by the STRING database (https://string-db.org/) and the targets with confidence score >0.95 were screened for network construction. As a result, 236 nodes and 666 edges were involved in this network. More crucial targets were obtained based on the degree value of network topology analysis. Among them, 69 targets with degree values greater than twice the median were considered as candidate targets (Supplementary Figure S2). Forty constituents that had direct effects on 69 candidate targets were screened out for further identified as the key active constituents of QYD in treating AP. Among them, 11 constituents including quercetin (**33**), wogonin (**97**), baicalein (**88**), emodin (**104**), epicatechin (**28**), aloe-Emodin (**93**), berberine (**71**), palmatine (**72**), oroxylin A (**101**), physcion (**108**) and skullcapflavon II (**100**) were considered to be the key active constituents because their degree value was more than twice the median value.

#### Analysis of Related Pathways of Potential Targets

**FIGURE 4:**
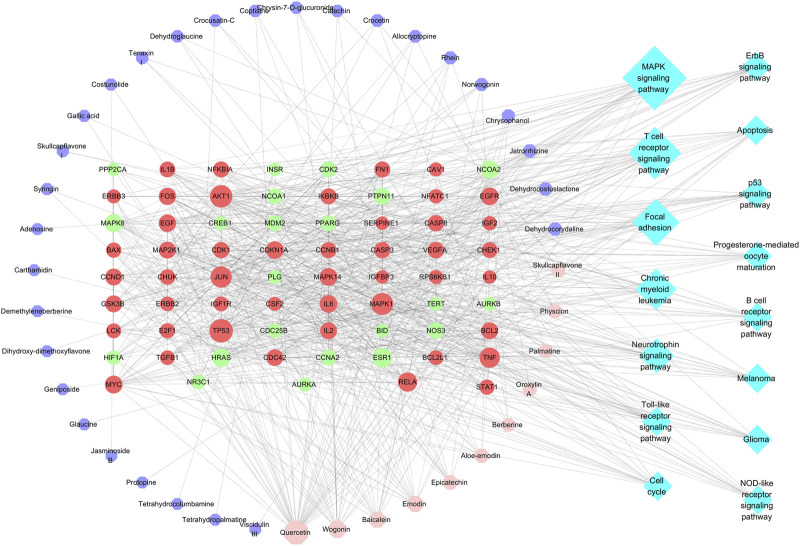
Compound-target-pathway network (Pink octagon represents the key compound; purple octagon represents common compound; red circles represents key target; green circles represents common target; blue diamond represents pathway. It’s a positive proportional relationship that between the node size and the degree).

DAVID website was used to carry out KEGG pathway enrichment analysis on potential targets and 63 pathways were obtained. Of which 59 were statistically significant (*p* < 0.05). Associations among active constituents, potential targets and the top 15 pathways (excepted cancer pathways) were shown in Figure 4. It could be seen that 11 key active ingredients could regulate relevant pathways by acting on 47 targets, and the MAPK signaling pathway, T cell receptor signaling pathway, Focal adhesion, Toll-like receptor signaling pathway and Apoptosis were involved. The details of their interactions were listed in Supplementary Table S4.

### Molecular Docking Analysis

**FIGURE 5:**
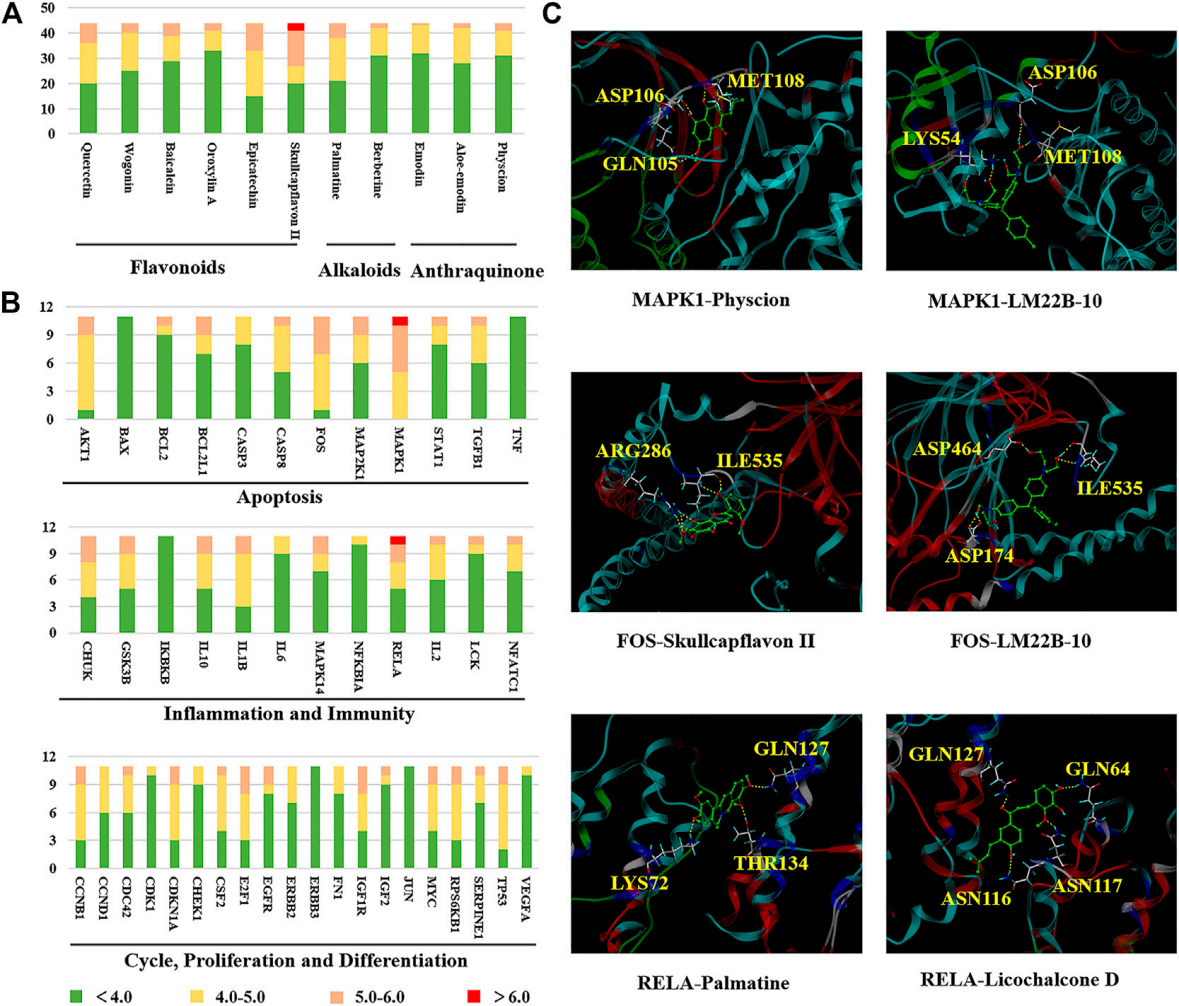
The binding modes of key compounds with related proteins were analyzed by SYBYL-X 2.0 software. **(A)** Component docking score; **(B)** Target docking score; **(C)** Representative docking combinations.

RCSB Protein Data Bank database was used to investigate the relationship between active constituents and potential targets by collecting the crystal structure of the target (Supplementary Table S5), and SYBYL-X 2.0 platform was applied to analyze the docking relationship. As shown in Figure 5A, the binding ability of components with targets were divided into three levels, which contain strong interaction (>6.0), medium interaction (5.0–6.0), weak interaction (4.0–5.0) and no interaction (<4.0). In this study, 5.0 was set as the screening value to obtain more critical targets. As a result, skullcapflavon II (a characteristic ingredient derived from *Scutellaria baicalensis* Georgi), palmatine (from *Corydalis yanhusuo* (Y. H. Chou and Chun C. Hsu) W. T. Wang ex Z. Y. Su and C. Y. Wu), and physcion (from *Rheum officinale* Baill*.*), showed better binding ability in flavonoids, alkaloids and anthraquinone compounds, respectively. As for the targets, MAPK1 (ERK, PDB ID: 6slg), FOS (c-Fos, PDB ID: 1a02), and RELA (p65, PDB ID: 4kv1) possess higher binding activity (Figure 5B). The representative three-dimensional patterns of docking combinations (MAPK1-physcion, FOS-skullcapflavon II, and RELA-palmatine) were shown in Figure 5C.

It was seen that there are four hydrogen bonds between MAPK1 and physcion (**108**), and three of which are the interaction between hydroxyl and MET108, ASP106 and GLN105, the other one is the interaction between MET108 and carbonyl. Three interactions consist in the combination of MAPK1 and the positive drug LM22B-10, namely MET108, ASP106 and LY654. In addition, in the binding pocket of FOS and skullcapflavon II (**100**), five hydrogen bonds formed by two amino acid residues (ARG286 and ILE535) and phenolic hydroxyl groups were found. Similarly, five hydrogen bonds were also found between FOS and the positive drug LM22B-10, which were formed by phenolic hydroxyl groups bond with ASP174, ASP464 and ILE535. For RELA, three hydrogen bonds were found connection with palmatine (**72**) with GLN127, LYS72 and THR134. In the docking combination of RELA and positive Licochalcone D, four hydrogen bonds formed by RELA and ASN116, ASN117, GLN127 and GLN64 were shown.

### Experimental Evaluation

#### Histopathological Examination and Biochemical Analysis

**FIGURE 6:**
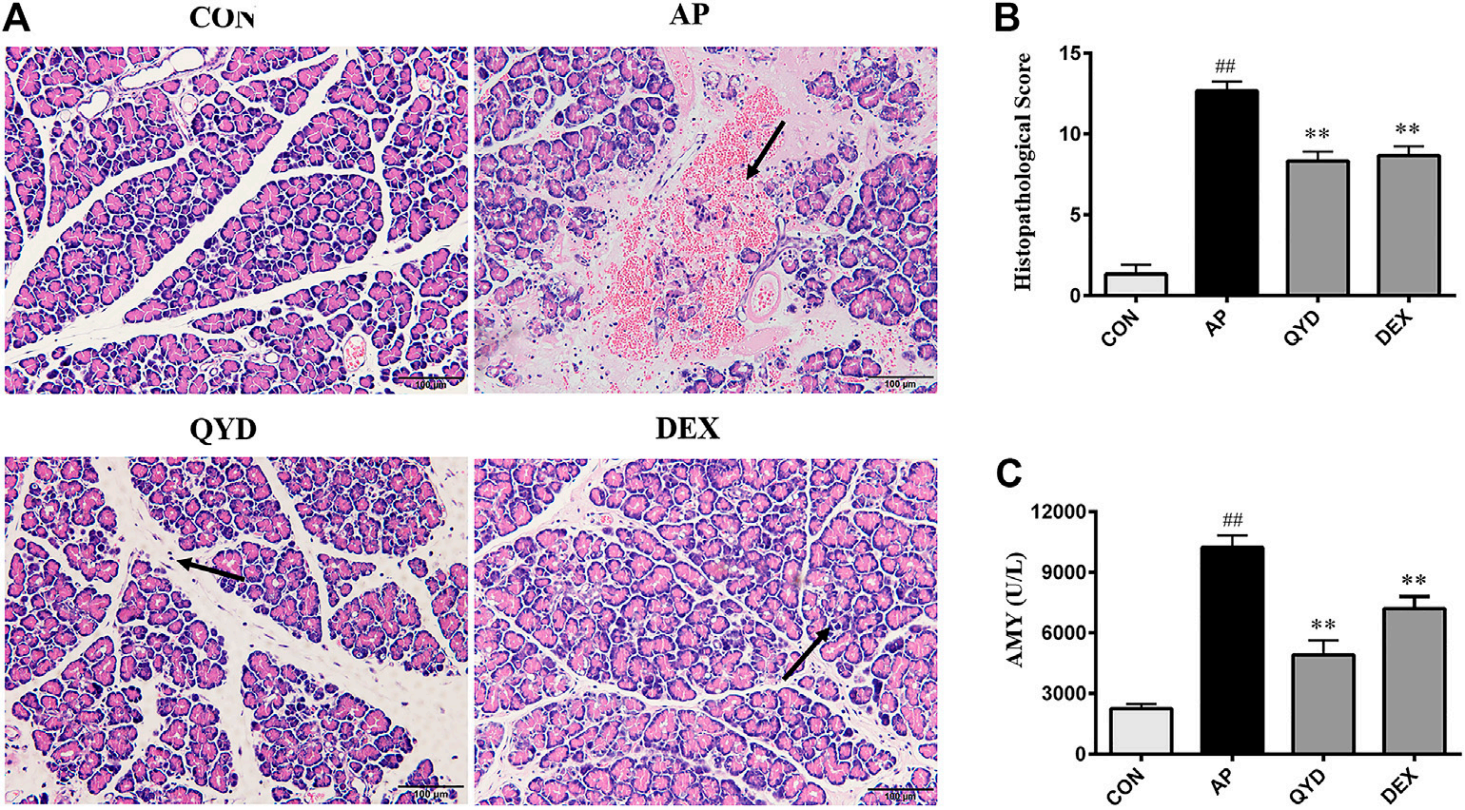
Histopathological examination and biochemical analysis. **(A)** Histopathological observation of pancreatic tissue in four groups (HE, ×200); **(B)** Histological score of pancreatic tissue in four groups (HE, ×200). **(C)** Serum amylase level in rats. Data are presented as the mean ± SD (*n* = 3), **p < 0.01 *vs.* AP group. ^##^ *p* < 0.01 *vs.* CON group.

In pancreatic tissue of AP rats, the acinar cell injury, vacuolation, hemorrhage and inflammatory cell infiltration occurred was observed. When compared with the AP group, different degrees of improvement in QYD and DEX treatment groups were seen, and the QYD group possess a better effect. As shown in Figure 6A, edema and hemorrhage were not observed between the cells; the structure of the pancreatic lobules was clear and without infiltrated by inflammatory cells in the CON group. For the AP group, obvious edema among histiocytes was observed, a large number of red blood cells was overflowed, and the pancreatic tissue was infiltrated by numerous neutrophils and lymphocytes. The structure of pancreatic lobules was fuzzy and uniform, and a large quantity of necrotic pancreatic cells was found. In the QYD group, there was mild edema among tissue cells, the pancreas was infiltrated by a small number of inflammatory cells, and some areas were calcified. As for the DEX group, there was edema between cells, the pancreas was infiltrated by a few inflammatory cells, and the infiltration was distributed in the lesion. Calcification was seen in some areas, and a small number of red blood cells are spilled around the blood vessels. Also, its histopathological score is shown in Figure 6B.

Besides, an abnormal increase of serum amylase level is an essential basis for the diagnosis of AP. In this experiment, the serum amylase level of rats was detected by the starch-iodine colorimetric method. The results showed (Figure 6C) that amylase levels in the AP group were significantly higher than those in the CON group (*p* < 0.01). In addition, compared with the AP group, QYD treatment could significantly inhibit the activity of amylase (*p* < 0.01).

### Western Blot Analysis

**FIGURE 7:**
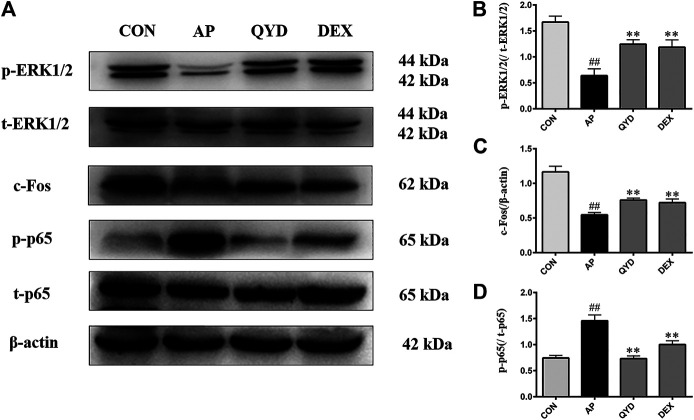
QYD extracts protect pancreas tissue of SD rats by regulated inflammation and cell apoptosis. **(A)** Effects of QYD extracts on p-p65, t-p65, p-ERK1/2, t-ERK1/2, c-Fos protein levels in STC-induced acute pancreatitis model based on the western blotting assay; **(B–D)** Statistical analysis of the effects of QYD extracts on protein expressions levels. Data are presented as the mean ± SD (*n* = 3), **p < 0.01 *vs.* AP group. ^##^ *p* < 0.01 *vs.* CON group.

The western blotting analysis was used to evaluate related protein levels in the pancreas. As shown in Figure 7, p-p65 expression was markedly up-regulated, and p-ERK1/2 and c-Fos expression were notably down-regulated in AP rats following STC infusion (*p* < 0.01). As expected, QYD and DEX treatment significantly increased p-ERK1/2 and c-Fos levels and decreased p-p65 levels compared to the AP group (*p* < 0.01), and QYD treatment group possesses a better effect. The results suggest that the therapeutic effect of QYD on AP may be related to inhibiting the expression of p-p65 and promoting the expression of p-ERK1/2 and c-Fos.

## Discussion

As a common critical disease of the digestive system, AP should attract our attention. Although the pathogenesis and clinical treatment of AP has been widely developed in recent basic and clinical research, satisfactory results are still not obtained. Therefore, it has become a significant challenge in clinical and scientific research to explore the mechanism of AP occurrence and development and provide new treatment suggestions for AP patients.

According to TCM theory, the pathogenesis of AP belongs to the category of Yang ming fu-viscera excess syndrome, which should be treated following the principles of soothing liver, regulating qi, clearing heat, detoxifying and removing stasis by purgation. QYD is developed based on the classic prescription Da-Cheng-Qi decoction of “Shang Han Lun”, which is composed of eight herbs and obtained a significant effect in the clinical treatment of AP. However, its material basis and the overall mechanism of action remain a mystery. In this study, a combined strategy of phytochemistry, network pharmacology, molecular docking and basic experiment was used to assess the active constituents and potential molecular mechanisms of QYD on AP step by step. Firstly, the chemical composition of QYD was characterized by UHPLC-QTOF-MS for the first time. A total of 110 constituents were identified, of which 47 conform to the Lipinski’s rule of five and mainly belong to alkaloids, flavonoids, and anthraquinones. Next, 11 key components were further filtered through network pharmacology, which contained six flavonoids that mainly from *Scutellaria baicalensis* Georgi [quercetin (**33**), wogonin (**97**), baicalein (**88**), epicatechin (**28**), oroxylin A (**101**), skullcapflavon II (**100**)], three anthraquinones that mainly from *Rheum officinale* Baill. [emodin (**104**), aloe-emodin (**93**), physcion (**108**)], and two alkaloids that mainly from *Corydalis yanhusuo* (Y. H. Chou and Chun C. Hsu) W. T. Wang ex Z. Y. Su and C. Y. Wu [berberine (**71**), palmatine (**72**)]. Interestingly, the effects of *Scutellaria baicalensis* Georgi, *Rheum officinale* Baill*.* and *Corydalis yanhusuo* (Y. H. Chou and Chun C. Hsu) W. T. Wang ex Z. Y. Su and C. Y. Wu in TCM are similar to the primary efficacy of QYD, which suggests that these three drugs play a significant role in QYD treatment of AP. This makes the prediction results of network pharmacology more convincing. Also, the main components of these three herbs have been preliminarily explored in the treatment of AP. Two previous studies found that baicalein (**88**) and quercetin (**33**) from *Scutellaria baicalensis* Georgi could alleviate the symptoms of AP by promoting cell apoptosis and inhibiting inflammation (Zheng et al., 2018; Pu et al., 2019). In addition, emodin (**104**) from *Rheum officinale* Baill*.* played an antioxidant and anti-inflammatory role in AP (Xia et al., 2019). Moreover, berberine (**71**) from *Corydalis yanhusuo* (Y. H. Chou and Chun C. Hsu) W. T. Wang ex Z. Y.Su and C. Y. Wu had analgesic effects, which involved relieving the pancreatic pain of QYD on AP (Choi et al., 2016). The above discussion fully affirmed the therapeutic potential of QYD in AP.

Apoptosis, inflammatory response, immune regulation, cell proliferation and differentiation are essential characteristics of AP development (Xiang et al., 2017). In this study, KEGG pathway enrichment analysis showed that 11 key components in the QYD regulate the information transmission of 15 signaling pathways by targeting 47 targets. Combined the results of KEGG and molecular docking, we found that the top targets in docking scores were near related to apoptosis and inflammation.

As is known to all, the AP’s development process is related to apoptosis and necrosis of pancreatic acinar cells, the MAPK signaling pathway and Apoptosis signaling pathway were enriched by apoptosis-related proteins such as AKT1, CASP3, FOS, etc. Our molecular docking results showed that a high overall score existed in MAPK1 (ERK1/2) and FOS (c-Fos) when docking with 11 key components, which suggested that they may play an essential role in QYD against AP. Previous studies have confirmed that ERK1/2 is a crucial target for MAPK signaling pathways. It is an important carrier that transfers transmitting signals from cell surface receptors to the nucleus, which could be activated by upstream signaling molecules and regulate corresponding biological functions like cell proliferation and apoptosis (Ma et al., 2013; Tanaka and Iino, 2015; Li et al., 2018b). Some other researchers have found that the process of apoptosis could be regulated by promoting phosphorylation of ERK1/2 (Damiano et al., 2018; Kim et al., 2019). Similarly, apoptosis could be promoted after c-Fos being activated, which is a downstream target of ERK1/2 (Pandey and Wang, 1995; Wagner, 2010; Ren et al., 2013). It has also been reported that accelerating apoptosis is beneficial to the disease remission in the early stage of AP because it delays the process of cell necrosis to some extent (Cai et al., 2018b) Consistent with this, our molecular biology experiments showed that there was a significantly decreased in the expression of ERK1/2 and c-Fos when AP occurred, while QYD restored their expression to some extent. Other critical proteins related to apoptosis, such as BCL-2 and caspase family, were also predicted (Morishima et al., 2002). It suggested that QYD treatment of AP may be partially achieved by promoting the process of apoptosis.

The NF-KB signaling pathway is one of the typical inflammatory response pathways and the inflammatory response is a typical feature of AP. Although this pathway did not show in the results of KEGG pathway enrichment analysis, it exists in the multiple signal pathways that have been enriched and play a vital role in them, such as MAPK signal pathway, T cell receptor signal pathway, Toll-like receptor signal pathway, and NOD-like receptor signaling pathway. RELA (p65) is an essential molecule in NF-KB signaling pathways who is responsible for the expression of proinflammatory mediators (Jakkampudi et al., 2016). When docking with critical components, it exhibited high binding activity. In some studies, inflammatory cascade reaction shows the importance to aggravate AP. The binding of p65 and IKB is inhibited under normal conditions. However, when something stimulates the cell, IKB will be degraded by IKK, p65 will be released and enter the nucleus quickly to binding with the DNA so that the transcription is initiated and a large number of inflammatory cells factors such as TNF-α, IL-6 and IL-1B are released. Then, the p65 is further activated, and the inflammatory response is gradually amplified (Hayden and Ghosh, 2008; Rotstein, 2014). Hence, we can see that inhibition of p65 expression could attenuate the severity of the inflammatory response and alleviate the symptoms of AP, which was also supported by the results of our experiment. In addition, other targets such as CHUK and GSK3B were also involved in inflammation and the AP process.

In recent years, more and more researches have focused on the human immune system. A study found that the disturbance of the immune regulation mechanism is one reason that aggravates the severity of AP and even leads to death (Kylänpää et al., 2010). AP could be improved by timely adjustment of the immunosuppressive state because it will urge a dynamic balance between the proinflammatory and anti-inflammatory responses (Munir et al., 2020). Other studies had shown that IL2, NFATC1 and LCK played a vital role in the T cell receptor signaling pathway. Among them, IL2 is necessary for T cell proliferation and other immune regulation, and it could stimulate B cells, monocytes and natural killer cells. And NFATC1 is very important for the induction of IL2 gene transcription. The differentiation and apoptosis of T cells was regulated by it (Chuvpilo et al., 1999). Besides, Lck is an src-related protein tyrosine kinase, which regulates T cells’ survival status by binding to CD4 molecules and the inflammatory response of AP (Bai et al., 2014; Bozso et al., 2020).

It is worth noting that among the targets predicted in this study, some proteins were related to the cell cycle, cell proliferation and cell differentiation. Among the multiple proteins, the CCNB1, CDC42, CDKN1A, E2F1, EGFR, MYC, and TP53 had a higher docking score. Previous studies believed that inflammation was closely related to immunity and the unfavorable supervision of the immune system is an advantage for tumorigenesis (Padoan et al., 2019). Meanwhile, repeated episodes of AP can lead to chronic pancreatitis, and scholars such as Kleeff J believed that chronic pancreatitis is an important risk factor for pancreatic ductal adenocarcinoma. Moreover, obvious binding effects between key components and tumor-related proteins suggested that QYD may have unexpected effects in preventing the transformation of pancreatitis from inflammation to cancer (Kleeff et al., 1965; Tarasiuk and Fichna 2019).

There are still two shortcomings in this study. On one hand, in terms of the research dimension, there is a lack of the validation from clinical and cellular molecular aspects. On the other hand, in terms of the research technology, network pharmacology ignores the dose-effect relationship of multi-components in formula when they exert the therapeutic effects. These deficiencies will be improved in subsequent studies.

**FIGURE 8:**
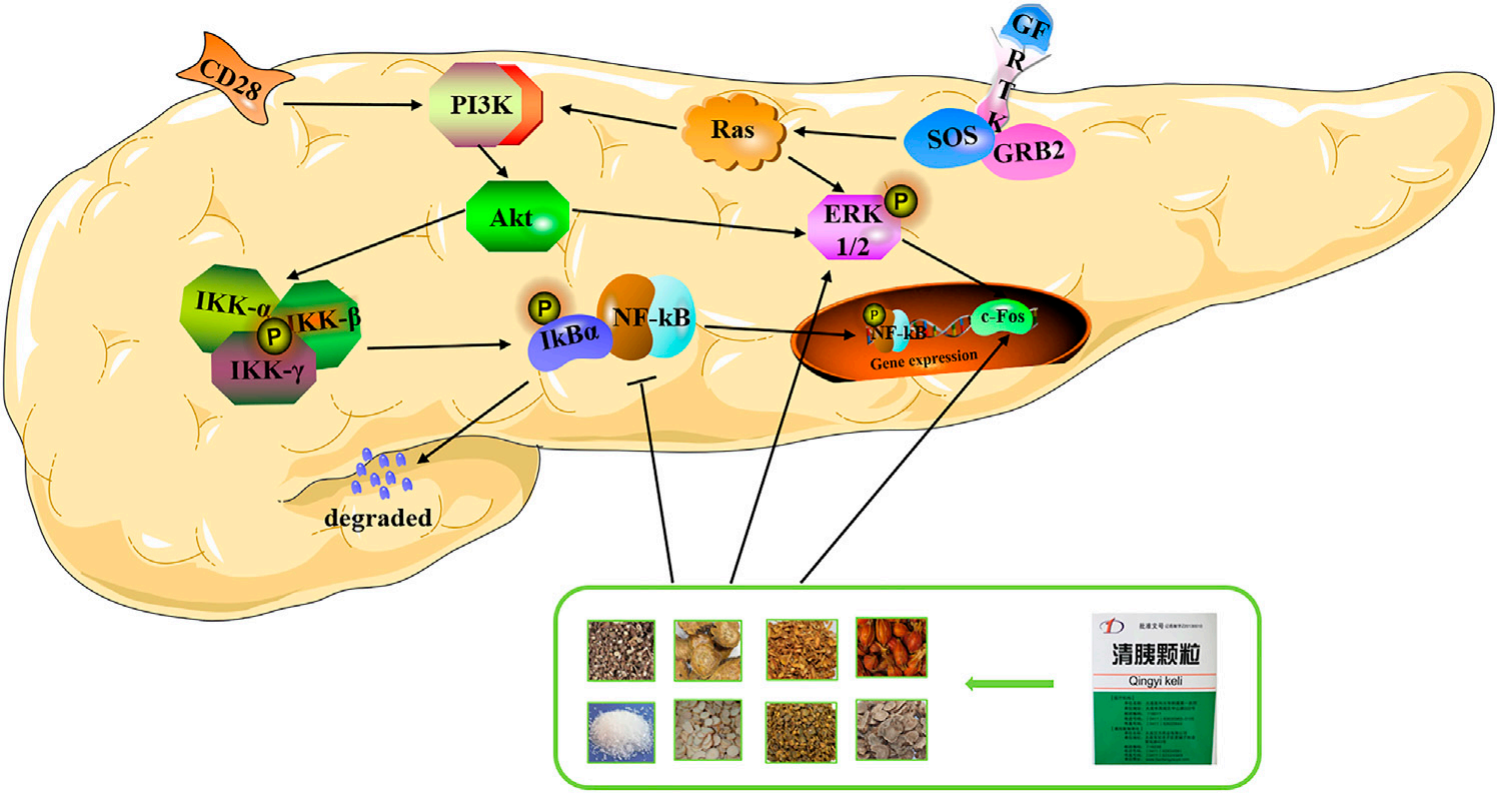
Overview of potential mechanisms underlying the protective effects of QYD on STC-induced acute pancreatitis.

In conclusion, our molecular biology experiments showed that QYD could reduce the expression of p-p65 and promote the expression of p-ERK1/2 and c-Fos significantly, which is consistent with the results of the prediction of network pharmacology and molecular docking. Based on this, we propose a plausible molecular mechanism for the multi-target effects of QYD on AP, namely, QYD may protect the pancreas from injury by promoting apoptosis and inhibiting inflammation (Figure 8).

## Conclusion

Chinese medicine plays a crucial role in preventing and treating AP. In this study, 110 chemical components of QYD were identified by UHPLC-QTOF-MS for the first time, and then 11 important components and 47 key targets were screened based on network pharmacological analysis. Molecular docking showed that QYD may influence the process of apoptosis and inflammation by regulating the expression of ERK1/2, c-Fos and p65, thus protecting pancreatic injury caused by AP. This result has been confirmed in molecular biology experiments. To sum up, our research not only provides a comprehensive understanding of the active components and molecular mechanism of QYD, but also provides experimental basis and new idea for further development and clinical application of QYD.

## Data Availability

The raw data supporting the conclusions of this article will be made available by the authors, without undue reservation, to any qualified researcher.
