# Supplementary material for: Qing-Yi Decoction in the Treatment of Acute Pancreatitis: An Integrated Approach Based on Chemical Profile, Network Pharmacology, Molecular Docking and Experimental Evaluation
Source: Front Pharmacol. 2021 Apr 29;12:590994. doi: 10.3389/fphar.2021.590994 (PMC8117095; doi:10.3389/fphar.2021.590994)
Supplement: Supplementary file 3 [file Table1.docx]

Table 1 Characterization of the chemical constituents in QYD by UHPLC–QTOF MS

Peak t_R_ Identification Formula Negative mode Positive mode Source *^a^*

No (min) Quasi-molecular Observed Calculated ppm Fragement ions *^c^* Quasi-molecular Observed Calculated ppm Fragement ions *^c^*

ion mass (Da) mass (Da) ion mass (Da) mass (Da)

1 1.56 Galloyl glucose C_13_H_16_O_10_ [M-H]^-^ 331.0672 331.0671 0.30 169.0119 [M-H-Glc] ^-^ — — — — — RRR

2 1.58 Adenosine C_10_H_13_N_5_O_4_ [M+HCOO] ^-^ 312.0946 312.0950 -1.28 — [M+H] ^+^ 268.1047 268.104 2.61 136.0622[M+H-C_5_H_8_O_4_] ^+^ PRA

119.0340[M+H-C_5_H_8_O_4_-NH_3_] ^+^

3 *^b^* 1.94 Gallic acid C_7_H_6_O_5_ [M-H] ^-^ 169.0145 169.0142 1.77 125.0246 [M-H-CO_2_] ^-^ [M+H] ^+^ 171.0293 171.0288 2.92 153.0169 [M+H-H_2_O] ^+^ RRR/PRA/GF

4 2.89 Phenylalanine C_9_H_11_NO_2_ [M-H] ^-^ 164.0723 164.0717 3.66 — [M+H] ^+^ 166.0867 166.0863 2.41 120.0811[M+H-CO_2_] ^+^ SR

120.0811[M+H-CH_3_NO_2_] ^+^

77.0390[M+H-C_3_H_6_NO_2_] ^+^

5 3.35 Gardoside C_16_H_22_O_10_ [M-H] ^-^ 373.1139 373.1140 -0.27 193.0515 [M-H-C_6_H_2_O_6_] ^-^ — — — — — GF

[M+Cl] ^-^ 409.0912 409.0907 1.22

6 3.80 Protocatechuic acid C_7_H_6_O_4_ [M-H] ^-^ 153.0194 153.0193 0.65 — — — — — — SR

7 3.98 6-O-L-d-Glucopyranosyl-lactinolide C_16_H_26_O_9_ [M-H] ^-^ 361.1510 361.1504 1.66 — [M+Na] ^+^ 385.1459 385.1469 -2.60 — PRA

[M+Cl] ^-^ 397.1275 397.1271 1.01

[M+HCOO] ^-^ 407.1558 407.1559 -0.25

8 4.16 Shanzhiside C_16_H_24_O_11_ [M+Cl] ^-^ 427.1014 427.1013 0.23 — — — — — — GF

[M-H] ^-^ 391.1247 391.1246 0.26

9 4.81 Deacetyl-asperulosidic acid methyl C_17_H_24_O_11_ [M+HCOO] ^-^ 449.1309 449.1301 1.78 241.0739 [M-H-Glc] ^-^ [M+Na] ^+^ 427.1202 427.1211 -2.11 265.0584 [M+H-Glc] ^+^ GF

ester [M+Cl] ^-^ 439.1019 439.1013 1.37 223.0647 [M-H-Glc] ^-^

[M-H] ^-^ 403.1239 403.1246 -1.74

10 5.11 Tryptophane C_11_H_12_N_2_O_2_ [M-H] ^-^ 203.0832 203.0826 2.95 — [M+H] ^+^ 205.0965 205.0972 -3.41 189.0497 [M+H-NH_2_] ^+^ RRR/PRA/GF/BR

119.0446[M+H-C_3_H_4_N_2_O_2_] ^+^

91.0495[M+H-C_4_H_6_N_2_O_2_] ^+^

11 5.31 Gardenoside C_17_H_24_O_11_ [M+HCOO] ^-^ 449.1300 449.1301 -0.22 241.0734 [M-H-Glc] ^-^ [M+Na] ^+^ 427.1209 427.1211 -0.47 265.0636 [M+H-Glc] ^+^ GF

[M+Cl] ^-^ 439.1021 439.1013 1.82 223.0631 [M-H-Glc] ^-^

[M-H] ^-^ 403.1253 403.1246 1.74

12 5.42 4-Hydroxybenzoic acid C_7_H_6_O_3_ [M-H] ^-^ 137.0232 137.0244 -8.76 — — — — — — SR

13 5.42 Jasminoside D C_16_H_26_O_8_ [M-H] ^-^ 345.1539 345.1555 -4.64 165.0925[M-H-Glc-H_2_O] ^-^ [M+H] ^+^ 347.1696 347.1700 -1.15 167.1011[M+H-C_6_H_10_O_5_-H_2_O] ^+^ GF

[M+Cl] ^-^ 381.1322 381.1325 -0.79 [M+Na] ^+^ 369.1517 369.1520 -0.81

[M+HCOO] ^-^ 391.1610 391.1610 0.00

14 5.70 Protocatechualdehyde C_7_H_6_O_3_ [M-H] ^-^ 137.0232 137.0244 -8.76 — — — — — — PRA

15 5.91 Scandoside methyl ester C_17_H_24_O_11_ [M+HCOO] ^-^ 449.1305 449.1301 0.89 241.0737 [M-H-Glc] ^-^ [M+Na] ^+^ 427.1210 427.1211 -0.23 — GF

[M+Cl] ^-^ 439.1014 439.1013 0.23

[M-H] ^-^ 403.1246 403.1246 0.00

16 6.13 Jasminoside B C_16_H_26_O_8_ [M-H] ^-^ 345.1556 345.1555 0.29 — [M+H] ^+^ 347.1696 347.1700 -1.15 185.1124[M+H-Glc] ^+^ GF

[M+Cl] ^-^ 381.1322 381.1325 -0.79 [M+Na] ^+^ 369.1513 369.1520 -1.90 167.1011[M+H-Glc-H_2_O] ^+^

[M+HCOO] ^-^ 391.1610 391.1610 0.00 GF

17 6.57 Jasminodiol C_10_H_16_O_3_ [M-H] ^-^ 183.1028 183.1027 0.55 — [M+H] ^+^ 185.1170 185.1172 -1.08 — GF

18 *^b^* 6.80 Catechin C_15_H_14_O_6_ [M-H] ^-^ 289.0729 289.0718 3.81 245.0831 [M-H-CO_2_] ^-^ [M+H] ^+^ 291.0860 291.0863 -1.03 — RRR/PRA

[M+Cl] ^-^ 325.0487 325.0484 0.92

19 *^b^* 6.95 Chlorogenic acid C_16_H_18_O_9_ [2M-H] ^-^ 707.1824 707.1829 -0.71 191.0565 [M-H-caffeoyl] ^-^ [M+Na] ^+^ 377.0843 377.0829 3.71 — GF

[M-H] ^-^ 353.0879 353.0878 0.28 [M+H] ^+^ 355.1021 355.1024 -0.84

20 7.04 Darendoside A C_19_H_28_O_11_ [M-H] ^-^ 431.1555 431.1559 -1.02 — [M+Na] ^+^ 455.1520 455.1524 -0.97 — SR

21 7.09 Oxypaeoniflorin C_23_H_28_O_12_ [M-H] ^-^ 495.1515 495.1508 1.41 289.0757 [M-H-C_15_H_9_O] ^-^ [M+Na] ^+^ 519.1474 519.1473 0.19 — PRA

22 *^b^* 7.30 Syringin C_17_H_24_O_9_ [M+HCOO] ^-^ 417.1401 417.1402 -0.24 — [M+Na] ^+^ 395.1310 395.1313 -0.76 — BR

[M+Cl] ^-^ 407.1116 407.1114 0.49

23 7.65 Hyperoside C_21_H_20_O_12_ [M-H] ^-^ 463.0884 463.0882 0.43 301.0349[M-H-Glc] ^-^ [M+H] ^+^ 465.1028 465.1028 0.00 303.0484[M-H-Glc]^+^ GF

283.0227[M-H-Glc-H_2_O] ^-^

257.0483[M-H-Glc-CO_2_] ^-^

175.0049[M-H-Glc-C_6_H_6_O_3_] ^-^

151.0044[M-H-Glc-C_8_H_6_O_3_] ^-^

125.0244[M-H-Glc-C_9_H_4_O_4_] ^-^

24 7.69 Genipin-1-β-gentiobioside C_23_H_34_O_15_ [M+HCOO] ^-^ 595.1884 595.1880 0.67 225.0784[M-H-gentiobiosyl] ^-^ [M+Na] ^+^ 573.1788 573.1790 -0.35 — GF

[M+Cl] ^-^ 585.1594 585.1592 0.34

[M-H] ^-^ 549.1822 549.1825 -0.55

25 8.61 Sinapyglucoside C_17_H_22_O_10_ [M-H] ^-^ 385.1146 385.1140 1.56 223.0607[M-H-Glc] ^-^ [M+Na] ^+^ 409.1104 409.1105 -0.24 — GF

205.0510[M-H-Glc-H_2_O] ^-^

26 8.77 Genipin C_11_H_14_O_5_ [M-H] ^-^ 225.0772 225.0768 1.78 123.0455 [M-H-C_4_H_6_O_3_] ^-^ [M+H] ^+^ 227.0922 227.0914 3.52 — GF

27 8.81 Geniposide C_17_H_24_O_10_ [M-H] ^-^ 387.1300 387.1297 0.77 225.0798[M-H-Glc] ^-^ [M+Na] ^+^ 411.1264 411.1262 0.49 — GF

[M+Cl] ^-^ 423.1065 423.1063 0.47 123.0457[M-H-C_10_H_16_O_8_] ^-^

[M+HCOO] ^-^ 433.1354 433.1351 0.69 101.0248[M-H-C_13_H_18_O_7_] ^-^

28 *^b^* 8.85 Epicatechin C_15_H_14_O_6_ [M-H] ^-^ 289.0715 289.0718 -1.04 — [M+H] ^+^ 291.0870 291.0863 2.40 — RRR

[M+Cl] ^-^ 325.0487 325.0484 0.92

29 *^b^* 9.59 Albiflorin C_23_H_28_O_11_ [M+HCOO] ^-^ 525.1624 525.1614 1.90 357.1170 [M-H-benzoic acid] ^-^ [M+Na] ^+^ 503.1502 503.1523 -4.17 319.1079[M+H-Glc]^+^ PRA

[M+Cl] ^-^ 515.1326 515.1326 0.00 283.0806 [M-H-C_16_H_21_O_9_] ^-^ [M+H] ^+^ 481.1690 481.1704 -2.91 301.0974[M+H-Glc-H_2_O] ^+^

[M-H] ^-^ 479.1555 479.1559 -0.83 121.0293 [M-H-C_16_H_21_O_9_] ^-^

30 10.36 Crocusatin-C C_10_H_16_O_2_ — — — — — [M+H] ^+^ 169.1219 169.1223 -2.37 151.1073 [M+H-H_2_O] ^+^ GF

107.0447 [M+H-C_3_H_10_O] ^+^

Table 1 Characterization of the chemical constituents in QYD by UHPLC–QTOF MS (*Continued*)

Peak t_R_ Identification Formula Negative mode Positive mode Source *^a^*

No (min) Quasi-molecular Observed Calculated ppm Fragement ions *^c^* Quasi-molecular Observed Calculated ppm Fragement ions *^c^*

ion mass (Da) mass (Da) ion mass (Da) mass (Da)

31 *^b^* 10.48 Paeoniflorin C_23_H_28_O_11_ [M+HCOO] ^-^ 525.1611 525.1614 -0.57 449.1454 [M-H-CH_2_O] ^-^ [M+Na] ^+^ 503.1499 503.1523 -4.77 PRA

[M+Cl] ^-^ 515.1325 515.1326 -0.19 327.1112 [M-H-CH_2_O-C_7_H_6_O_2_] ^-^

[M-H] ^-^ 479.1562 479.1559 0.63 165.0558 [M-H-C_14_H_18_O_8_] ^-^

32 11.04 Jasminoside C C_16_H_24_O_7_ [M+HCOO] ^-^ 373.1502 373.1504 -0.54 121.0300 [M-H-C_16_H_21_O_9_] ^-^ [M+H] ^+^ 329.1574 329.1595 -6.38 — GF

[M+Cl] ^-^ 363.1219 363.1216 0.83

33 11.34 Quercetin C_15_H_10_O_7_ [M-H] ^-^ 301.0360 301.0354 1.99 257.0476 [M-H-CO_2_] ^-^ [M+H] ^-^ 303.0501 303.0499 0.66 — BR

151.0038 [M-H-C_8_H_6_O_3_] ^-^

125.0244 [M-H-C_9_H_4_O_4_] ^-^

34 11.52 2-Methyl-l-erythritol-4-O-(6-O- C_22_H_32_O_13_ [M-H] ^-^ 503.1766 503.1770 -0.79 — [M+Na] ^+^ 527.1737 527.1735 0.38 — GF

transsinapoyl)-β-d-glucopyranoside

35 *^b^* 13.00 Rutin C_27_H_30_O_16_ [M-H] ^-^ 609.1460 609.1461 -0.16 301.0346 [M-H-rutinosyl] ^-^ [M+H] ^-^ 611.16 611.1607 0.49 303.0471 [M-H-rutinosyl] ^+^

36 *^b^* 13.42 Scutellarin C_21_H_18_O_12_ [M-H] ^-^ 461.0700 461.0725 0.21 285.0415[M-H-GluA] ^-^ [M+H] ^+^ 463.09 463.0871 -1.08 — SR

271.0251[M-H-GluA-CH_2_] ^-^

243.0299[M-H-GluA-CH_2_-CO] ^-^

37 13.38 Tetrahydrocolumbamine C_20_H_23_NO_4_ — — — — — [M+H] ^+^ 342.1701 342.1699 0.58 178.0871 [RDA C-ring opening] ^+^ CR

38 13.43 Isoquercitrin C_21_H_20_O_12_ [M-H] ^-^ 463.0882 463.0882 0.00 301.0349[M-H-Glc] ^-^ [M+H] ^+^ 465.1033 465.1028 1.08 303.0443[M+H-Glc] ^+^ GF

39 13.58 Glaucine C_21_H_25_NO_4_ — — — — — [M+H] ^+^ 356.1852 356.1856 -1.12 192.1008 [RDA C-ring opening] ^+^ CR

177.0762 [RDA-CH_3_] ^+^

40 14.41 Acteoside C_29_H_36_O_15_ [M-H] ^-^ 623.1984 623.1981 0.4814 461.1699[M-H-C_9_H_6_O_3_] ^-^ [M+Na] ^+^ 647.1937 647.1946 -1.33 — SR

315.1090[M-H-C_9_H_6_O_3_-(Rha-H_2_O)] ^-^

161.0244[M-H-C_20_H_30_O_12_] ^-^

133.0285[M-H-C_21_H_30_O_13_] ^-^

41 14.54 Protopine C_20_H_19_NO_5_ — — — — — [M+H] ^+^ 354.1339 354.1336 0.85 206.0815, 149.0599 [RDA C-ring opening] ^+^ CR

188.0710 [RDA-H_2_O] ^+^

42 15.45 (+)-Corybulbine C_21_H_25_NO_4_ — — — — — [M+H] ^+^ 356.1852 356.1856 -1.12 151.0693 [RDA C-ring opening] ^+^ CR

— 178.0803 [RDA C-ring opening-2×CH_2_] ^+^

43 16.05 Allocryptopine C_21_H_23_NO_5_ — — — — — [M+H] ^+^ 370.1647 370.1649 -0.54 206.790, 165.0886[RDA C-ring opening] ^+^ CR

188.0683 [RDA-H_2_O] ^+^

44 16.30 Demethyleneberberine C_19_H_17_NO_4_ — — — — — [M+H] ^+^ 324.1234 324.1230 1.23 176.0646, 149.0535 [RDA C-ring opening] ^+^ CR

45 16.38 Jasminoside S/H/I C_22_H_36_O_12_ [M+HCOO] ^-^ 537.2170 537.2189 -3.54 — [M+Na] ^+^ 515.2091 515.2099 -1.55 — GF

[M+Cl] ^-^ 527.1898 527.1901 -0.57

[M-H] ^-^ 491.2127 491.2134 -1.43

46 16.52 Coptisine C_19_H_14_NO_4_^+^ — — — — — [M] ^+^ 320.0912 320.0917 -1.56 292.0924[M-CO] ^+^ CR

277.0692[M-CO-CH_3_] ^+^

47 *^b^* 16.55 Tetrahydropalmatine C_21_H_25_NO_4_ — — — — — [M+H] ^+^ 356.1889 356.1856 9.26 192.1025 [RDA C-ring opening] ^+^ CR

48 *^b^* 16.72 Jatrorrhizine C_20_H_20_NO_4_^+^ — — — — — [M] ^+^ 338.1395 338.1387 2.37 323.1099{M-CH_3_] ^+^ CR

49 16.72 3,5-Dicaffeoylquinic acid/ C_25_H_24_O_12_ [M-H] ^-^ 515.1209 515.1195 2.72 353.0907 [M-H-C_9_H_6_O_3_] ^-^ [M+Na] ^+^ 539.1167 539.1160 -0.56 — GF

3,4-Dicaffeoylquinic acid 191.0564 [M-H-C_18_H_13_O_6_] ^-^

50 16.80 Mudanpioside I C_23_H_28_O_11_ [M+HCOO] ^-^ 525.1607 525.1614 -1.33 121.0295 [M-H-C_16_H_21_O_9_] ^-^ [M+Na] ^+^ 503.1501 503.1523 -4.37 — PRA

[M+Cl] ^-^ 515.1273 515.1326 -10.29 [M+H] ^+^ 481.1698 481.1704 -1.25

[M-H] ^-^ 479.1557 479.1559 -0.42

51 17.01 Leucosceptoside A C_30_H_38_O_15_ [M-H] ^-^ 637.2125 637.2138 -2.04 461.1707[M-H-C_10_H_8_O_3_] ^-^ — — — — — SR

[M+Cl] ^-^ 673.1897 673.1905 -1.19

52 17.29 6″-O-trans-p-Coumaroylgenipin C_32_H_40_O_17_ [M-H] ^-^ 695.2186 695.2193 -1.01 469.1327[M-H-C_11_H_14_O_5_] ^-^ [M+Na] ^+^ 719.2160 719.2158 0.28 — GF

[M+Cl] ^-^ 731.1969 731.1960 1.23 225.0775[M-H-C_21_H_26_O_12_] ^-^

207.0652[M-H-C_21_H_28_O_13_] ^-^

123.044[M-H-C_25_H_32_O_15_] ^-^

101.0242[M-H-C_28_H_34_O_16_] ^-^

53 17.60 Canadine C_20_H_21_NO_4_ — — — — — [M+H] ^+^ 340.1548 340.1543 1.47 149.0601 [RDA C-ring opening] ^+^ CR

176.0714[RDA-O] ^+^

54 17.73 Carthamidin C_15_H_12_O_6_ [M-H] ^-^ 287.0562 287.0561 0.42 — [M+H] ^+^ 289.0704 289.0707 -1.04 153.0118[RDA C-ring opening] ^+^ SR

55 17.76 6''-O-trans-Sinapoyl genipin C_34_H_44_O_19_ [M-H] ^-^ 755.2384 755.2404 -2.65 529.1510[M-H-C_4_H_18_O_10_] ^-^ [M+Na] ^+^ 779.2362 779.2369 -0.90 571.1562[M+H-C_2_H_18_O_9_] ^+^ GF

gentiobioside [M+Cl] ^-^ 791.2165 791.2171 -0.76 553.1508[M+H-C_2_H_18_O_9_] ^+^

56 17.80 3,5-di-O-Caffeoyl-4-O-(3-hydroxy- C_31_H_32_O_16_ [M-H] ^-^ 659.1620 659.1618 0.30 497.1293[M-H-caffeoyl] ^-^ [M+Na] ^+^ 683.1581 683.1583 -0.29 — GF

3-methyl)-glutaroylquinic acid 353.0897[M-H-C_15_H_14_O_7_] ^-^

335.0865[M-H-C_11_H_16_O_11_] ^-^

233.0647[M-C_15_H_22_O_14_] ^-^

191.0563[M-C_24_H_20_O_10_] ^-^

161.0473[M-H-C_25_H_22_O_11_] ^-^

57 17.81 Viscidulin III C_17_H_14_O_8_ [M-H] ^-^ 345.0616 345.0616 0.00 330.0385[M-H-CH_3_] ^-^ [M+H] ^+^ 347.0754 347.0761 -2.02 — SR

315.0136[M-H-2×CH_3_] ^-^

58 17.99 5,7,2'-Trihydroxy-6-methoxyflavone C_22_H_22_O_11_ [M-H] ^-^ 461.1093 461.1089 0.87 285.0405[M-H-Glc] ^-^ [M+H] ^+^ 463.1222 463.1235 -2.81 — SR

7-O-β-D-glucoside

59 17.98 Dehydroglaucine C_21_H_23_NO_4_ — — — — — [M+H] ^+^ 354.1699 354.1699 0.00 190.0777, 165.0854[RDA C-ring opening] ^+^ CR

60 18.24 Corydaline C_22_H_27_NO_4_ — — — — — [M+H] ^+^ 370.2018 370.2012 1.62 165.0912[RDA C-ring opening] ^+^ CR

192.1020{RDA-CH_2_] ^+^

Table 1 Characterization of the chemical constituents in QYD by UHPLC–QTOF MS (*Continued*)

Peak t_R_ Identification Formula Negative mode Positive mode Source *^a^*

No (min) Quasi-molecular Observed Calculated ppm Fragement ions *^c^* Quasi-molecular Observed Calculated ppm Fragement ions *^c^*

ion mass (Da) mass (Da) ion mass (Da) mass (Da)

61 18.48 Jasminoside T C_21_H_34_O_11_ [M+HCOO] ^-^ 507.2081 507.2083 -0.39 — [M+Na] ^+^ 485.1989 485.1993 -0.82 — GF

62 *^b^* 18.64 Baicalin C_21_H_18_O_11_ [M-H] ^-^ 445.0775 445.0776 -0.31 269.0461[M-H-GluA] ^-^ [M+H] ^+^ 447.0925 447.0922 0.67 271.0507[M+H-GluA] ^+^ SR

[2M-H] ^-^ 891.1630 891.1625 0.56 [M+Na] ^+^ 469.0738 469.0741 -0.64

63 18.70 Crocetin C_20_H_24_O_4_ — — — — — [M+H] ^+^ 329.1730 329.1747 -5.16 — GF

64 18.71 Crocin-I C_44_H_64_O_24_ [M-H] ^-^ 975.3697 975.3715 -1.85 651.266[M-H-C_6_H_20_O_10_] ^-^ — — — — — GF

[M+Cl] ^-^ 1011.3491 1011.3482 0.89 327.1623[M-H-C_21_H_36_O_15_] ^-^

65 18.74 Baicalein 7-O-β-D-glucoside C_21_H_20_O_10_ [M-H] ^-^ 431.0984 431.0984 0.05 269.0468[M-H-Glc] ^-^ [M+H] ^+^ 433.1133 433.1129 0.92 271.0507[M+H-GluA] ^+^ SR

251.0372[M-H-H_2_O] ^-^

241.0492[M-H-CO] ^-^

223.0355[M-H-CO-H_2_O] ^-^

66 18.86 6'-O-trans-Sinapoyl jasminoside L C_27_H_36_O_12_ [M-H] ^-^ 551.2126 551.2134 -1.45 533.2027[M-H-H_2_O] ^-^ [M+H] ^+^ 553.2278 553.2280 -0.36 — GF

[M+Cl] ^-^ 587.1895 587.1901 -1.02 521.2033[M-H-CH_2_O] ^-^ [M+Na] ^+^ 575.2112 575.2099 2.26

367.0984[M-H-C_10_H_15_O_3_] ^-^

67 18.93 Viscidulin II 2'-O-β-D-glucuronide C_23_H_22_O_13_ [M-H] ^-^ 505.0986 505.0988 -0.40 329.0673[M-H-GluA] ^-^ [M+H] ^+^ 507.1135 507.1133 0.39 331.0753[M+H-GluA] ^+^ SR

316.0504[M+H-GluA-CH_3_] ^+^

301.0269[M+H-GluA-2×CH_3_] ^+^

68 19.15 3-O-Caffeoyl-4-O-sinapoylquinic C_27_H_28_O_13_ [M-H] ^-^ 559.1453 559.1457 -0.72 397.1143 [M-H-caffeoyl] ^-^ [M+Na] ^+^ 583.1424 583.1422 0.41 421.1025 [M+Na-caffeoyl] ^+^ GF

acid 223.0603 [M-H-caffeoyl-C_7_H_10_O_5_] ^-^

173.0458 [M-H-sinapoylquinic acid] ^-^

69 19.48 Viscidulin III 6-O-β-D-glucoside C_23_H_22_O_13_ [M-H] ^-^ 505.0985 505.0988 -0.59 — [M+H] ^+^ 507.1135 507.1133 0.39 331.0780[M+H-GluA] ^+^ SR

316.0516[M+H-GluA-CH_3_] ^+^

301.0288[M+H-GluA-2×CH_3_] ^+^

70 19.68 Dihydrobaicalin C_21_H_20_O_11_ [M-H] ^-^ 447.0926 447.0933 -1.57 — [M+H] ^+^ 449.1073 449.1078 -1.11 — SR

[M+Na] ^+^ 471.0885 471.0898 -2.76

71 19.73 Berberine C_20_H_18_NO_4_^+^ — — — — — [M] ^+^ 336.1229 336.1230 -0.30 320.0897[M-CH_3_]+ CR

292.0949[M-CH_3_-CO] ^+^

72 *^b^* 19.75 Palmatine C_21_H_22_NO_4_^+^ — — — — — [M] ^+^ 352.1548 352.1543 1.42 336.1153[M-CH_3_] ^+^ CR

308.1201[M-CH_3_-CO] ^+^

73 20.30 Norwogonin 7-O-β-D-glucuronide C_21_H_18_O_11_ [M-H] ^-^ 445.0778 445.0776 0.34 269.0462[M-H-GluA] ^-^ [M+H] ^+^ 447.0926 447.0922 0.89 271.0506[M+H-GluA] ^+^ SR

74 20.30 Cistanoside D C_31_H_40_O_15_ [M-H] ^-^ 651.2284 651.2294 -1.54 351.0567[M-H-C_15_H_8_O_7_] ^-^ [M+Na] ^+^ 675.2258 675.2259 -0.21 — GF

[M+Cl] ^-^ 687.2050 687.2061 -1.60 175.0395[M-H-C_21_H_32_O_12_] ^-^

75 20.82 Crocin-2 C_38_H_54_O_19_ [M-H] ^-^ 813.3187 813.3187 0.00 651.2700[M-H-C_3_H_12_O_5_] ^-^ [M+Na] ^+^ 837.3156 837.3152 0.48 513.2081[M+H-C_10_H_22_O_10_] ^+^ GF

327.1627[M-H-C_18_H_28_O_10_] ^-^ 347.0922[M+H-C_24_H_36_O_9_] ^+^

76 20.92 5,6,7-Trihydroxy-8-methoxy C_22_H_20_O_12_ [M-H] ^-^ 475.0880 475.0882 -0.44 299.0557[M-H-GluA] ^-^ [M+H] ^+^ 477.1029 477.1028 0.15 301.0707[M+H-GluA] ^+^ SR

flavone 7-O-glucuronide 284.0323[M-H-GluA-CH_3_] ^-^ [M+Na] ^+^ 499.0842 499.0847 -1.00

77 21.27 Dehydrocorydaline C_22_H_24_NO_4_^+^ — — — — — [M] ^+^ 366.1719 366.1699 5.46 350.1308[M-CH_3_] ^+^ CR

322.1354[M-CH_3_-CO] ^+^

78 21.44 Chrysin-7-O-glucuronide C_21_H_18_O_10_ [M-H] ^-^ 429.0836 429.0827 2.10 253.0499[M-H-GluA] ^-^ [M+H] ^+^ 431.0983 431.0973 2.25 255.0614[M+H-GluA] ^+^ SR

79 21.55 isomer of Cistanoside D C_31_H_40_O_15_ [M-H] ^-^ 651.2288 651.2294 -0.95 351.0539[M-H-C_15_H_8_O_7_] ^-^ [M+Na] ^+^ 675.2256 675.2259 -0.50 GF

[M+Cl] ^-^ 687.2045 687.2061 -2.33 193.052[M-H-C_21_H_32_O_12_] ^-^

175.0412[M-H-C_21_H_32_O_12_] ^-^

80 21.61 Oroxyloside C_22_H_20_O_11_ [M-H] ^-^ 459.0938 459.0933 1.09 283.0615[M-H-GluA] ^-^ [M+H] ^+^ 461.1072 461.1078 -1.30 285.0692[M+H-GluA] ^+^ SR

175.0401[M-H-GluA-C_6_H_5_O_2_] ^-^ [M+Na] ^+^ 483.0893 483.0898 -1.04

81 22.66 Wogonoside C_22_H_20_O_11_ [M-H] ^-^ 459.0938 459.0933 1.09 283.0615[M-H-GluA] ^-^ [M+H] ^+^ 461.1072 461.1078 -1.30 285.0706[M+H-GluA] ^+^ SR

268.0380[M-H-GluA-CH_3_] ^-^ [M+Na] ^+^ 483.0893 483.0898 -1.04 270.0470[M+H-GluA-CH_3_] ^+^

82 23.23 5,7-Dihydroxy-8,2'-dimethoxy- C_23_H_22_O_12_ [M-H] ^-^ 489.1040 489.1038 0.41 313.0723[M-H-GluA] ^-^ [M+H] ^+^ 491.1186 491.1184 0.39 315.0815[M+H-GluA] ^+^ SR

flavone 7-O-β-D-glucuronide 298.0485[M-H-GluA-CH_3_] ^-^ [M+Na] ^+^ 513.0999 513.1003 -0.78 300.0572[M+H-GluA-CH_3_] ^+^

283.0252[M-H-GluA-2×CH_3_] ^-^ 285.0349[M+H-GluA-2×CH_3_] ^+^

83 24.12 Benzoyloxyalbiflorin C_30_H_32_O_12_ [M+HCOO] ^-^ 629.1878 629.1876 0.32 553.1740 [M-H-CHO] ^-^ [M+H] ^+^ 585.1964 585.1967 -0.51 — PRA

[M+Cl] ^-^ 619.1589 619.1588 0.16 431.1382 [M-H-CHO-C_7_H_6_O_2_] ^-^ [M+Na] ^+^ 607.1795 607.1786 1.48

[M-H] ^-^ 583.1853 583.1821 5.49 121.0296 [M-H-C_23_H_25_O_10_] ^-^

84 24.37 Norwogonin C_15_H_10_O_5_ [M-H] ^-^ 269.0455 269.0455 0.00 241.0491[M-H-CO] ^-^ [M+H] ^+^ 271.0597 271.0601 -1.48 — SR

225.0555[M-H-CO-O] ^-^

213.0498[M-H-2×CO] ^-^

85 *^b^* 24.41 Benzoylpaeoniflorin C_30_H_32_O_12_ [M+HCOO] ^-^ 629.1878 629.1876 0.32 553.1732 [M-H-CHO] ^-^ [M+H] ^+^ 585.1959 585.1967 -1.37 — PRA

[M+Cl] ^-^ 619.1587 619.1588 -0.16 121.0292 [M-H-C_23_H_25_O_10_] ^-^ [M+Na] ^+^ 607.1787 607.1786 0.16

[M-H] ^-^ 583.1840 583.1821 3.26

86 24.85 Tenaxin II C_16_H_12_O_6_ [M-H] ^-^ 299.0570 299.0561 3.01 284.0335[M-H-CH_3_] ^-^ [M+H] ^+^ 301.0717 301.0707 3.32 286.0406[M+H-CH_3_] ^+^ SR

258.0440[M+H-CH_3_-CO] ^+^

229.0478[M+H-CH_3_-CO-CHO] ^+^

87 25.04 Crocin-3 C_32_H_44_O_14_ [M-H] ^-^ 651.2646 651.2658 -1.89 327.161[M-H-C_12_H_20_O_10_] ^-^ [M+Na] ^+^ 675.2626 675.2623 0.40 347.0914[M+H-C_11_H_30_O_9_]^-^ GF

[M+Cl] ^-^ 687.2431 687.2425 0.87 283.1700[M-H-C_12_H_20_O_10_-CO_2_] ^-^

[M+HCOO] ^-^ 697.2709 697.2713 -0.57

88 *^b^* 25.07 Baicalein C_15_H_10_O_5_ [M-H] ^-^ 269.0455 269.0455 0.00 251.0356[M-H-H_2_O] ^-^ [M+H] ^+^ 271.0597 271.0601 -1.48 153.0144[M+H-RDA]^+^ SR

241.0508[M-H-CO] ^-^

223.0399[M-H-CO-H_2_O] ^-^

Table 1 Characterization of the chemical constituents in QYD by UHPLC–QTOF MS (*Continued*)

Peak t_R_ Identification Formula Negative mode Positive mode Source *^a^*

No (min) Quasi-molecular Observed Calculated ppm Fragement ions *^c^* Quasi-molecular Observed Calculated ppm Fragement ions *^c^*

ion mass (Da) mass (Da) ion mass (Da) mass (Da)

89 26.05 Laccaic acid D-8-O-(6′-O- C_31_H_26_O_13_ [M-H] ^-^ 605.1291 605.1301 -1.65 311.0567[M-H-C_14_H_14_O_7_] ^-^ [M+Na] ^+^ 629.1256 629.1266 -1.59 — RRR

cinnamoyl)-glucopyranoside 269.0454[M-C_16_H_16_O_8_] ^-^

90 26.08 Saikosaponin c or Saikosaponin BK1 C_48_H_78_O_17_ [M-H] ^-^ 925.5172 925.5166 0.65 779.4568[M-H-Rha] ^-^ [M+Na] ^-^ 949.5119 949.5131 -1.26 — BR

[M+Cl] ^-^ 961.4933 961.4933 0.00 617.4018[M-H-Rha-Glc] ^-^

[M+HCOO] ^-^ 971.5233 971.5221 1.24

91 26.29 Saikosaponin f C_48_H_80_O_17_ [M-H] ^-^ 927.5317 927.5323 -0.65 781.4736[M-H-Rha] ^-^ [M+Na] ^-^ 951.5293 951.5288 0.53 — BR

[M+Cl] ^-^ 963.5099 963.5090 0.93 765.4776[M-H-Rha-O] ^-^

[M+HCOO] ^-^ 973.5385 973.5378 0.72 619.4239[M-H-Rha-Glc] ^-^

92 26.55 Saikosaponin c or Saikosaponin BK1 C_48_H_78_O_17_ [M-H] ^-^ 925.5141 925.5166 -2.70 779.4543[M-H-Rha] ^-^ [M+Na] ^-^ 949.5127 949.5131 -0.42 — BR

[M+Cl] ^-^ 961.4940 961.4933 0.73 617.4081[M-H-Rha-Glc] ^-^

[M+HCOO] ^-^ 971.5220 971.5221 -0.10

93 *^b^* 26.83 Aloe-emodin C_15_H_10_O_5_ [M-H] ^-^ 269.0460 269.0455 1.86 — [M+H] ^+^ 271.0609 271.0601 2.95 253.0465[M+H-H_2_O] ^+^ RRR

241.0476[M+H-CH_2_O] ^+^

225.0506[M+H-CH_2_O_2_] ^+^

94 *^b^* 27.54 Rhein C_15_H_8_O_6_ [M-H] ^-^ 283.0253 283.0248 1.77 239.0338[M-H-CO_2_] ^-^ [M+H] ^+^ 285.0400 285.0394 2.10 267.0250[M+H-H_2_O] ^+^ RRR

211.0394[M-H-CO_2_-CO] ^-^ 241.0469[M+H-CO_2_] ^+^

183.0464[M-H-CO_2_-2×CO] ^-^

95 27.86 Skullcapflavone C_18_H_16_O_7_ [M-H] ^-^ 343.0821 343.0823 -0.58 328.0555[M-H-CH_3_] ^-^ [M+H] ^+^ 345.0966 345.0969 -0.87 330.0724[M+H-CH_3_] ^+^ SR

313.0336[M-H-2×CH_3_] ^-^ 315.0463[M+H-2×CH_3_]^+^

96 *^b^* 28.02 Saikosaponin A C_42_H_68_O_13_ [M-H] ^-^ 779.4576 779.4587 -1.41 617.4062[M-H-Glc] ^-^ [M+Na] ^-^ 803.4559 803.4552 0.87 — BR

[M+Cl] ^-^ 815.4356 815.4354 0.25 471.3509[M-H-Glc-Fuc] ^-^

[M+HCOO] ^-^ 825.4646 825.4642 0.48

97 *^b^* 28.02 Wogonin C_16_H_12_O_5_ [M-H] ^-^ 283.0619 283.0612 2.47 268.0384[M-H-CH_2_] ^-^ [M+H] ^+^ 285.0759 285.0757 0.70 255.0630[M+H-CH_2_O] ^+^ SR

253.0514[M-H-CH_2_O] ^-^

98 28.36 Dihydroxy-dimethoxyflavone C_17_H_14_O_6_ [M-H] ^-^ 313.0719 313.0718 0.32 285.0766[M-H-CO] ^-^ [M+H] ^+^ 315.0860 315.0863 -0.98 300.0606[M+H-CH_3_] ^+^ SR

283.0617[M-H-CH_2_O] ^-^ 285.0368[M+H-2×CH_3_] ^+^

269.0453[M-H-C_2_H_3_O] ^-^ 257.0419[M+H-2×CH_3_-CHO] ^+^

99 28.58 Saikosaponin B2 C_42_H_68_O_13_ [M-H] ^-^ 779.4588 779.4587 0.13 617.4066[M-H-Glc] ^-^ [M+Na] ^-^ 803.4542 803.4552 -1.24 — BR

[M+Cl] ^-^ 815.4357 815.4354 0.37 471.3517[M-H-Glc-Fuc] ^-^

[M+HCOO] ^-^ 825.4655 825.4642 1.57

100 28.63 Skullcapflavon II C_19_H_18_O_8_ [M-H] ^-^ 373.0936 373.0929 1.88 358.0670[M-H-CH_3_] ^-^ [M+H] ^+^ 375.1072 375.1074 -0.53 360.0760[M+H-CH_3_] ^+^ SR

343.0461[M-H-2×CH_3_] ^-^ 345.0546[M+H-2×CH_3_] ^+^

327.0419[M+H-2×CH_3_-H_2_O] ^+^

101 28.67 Oroxylin A C_16_H_12_O_5_ [M-H] ^-^ 283.0621 283.0612 3.18 268.0385[M-H-CH_3_] ^-^ [M+H] ^+^ 285.0773 285.0757 5.61 270.0453[M+H-CH_3_] ^+^ SR

242.0525[M+H-CH_3_-CO] ^+^

168.0003[M+H-CH_3_-RDA] ^+^

140.0054[M+H-CH_3_-RDA-CO] ^+^

102 28.81 Saikosaponin B1 C_42_H_68_O_13_ [M-H] ^-^ 779.4584 779.4587 -0.38 617.4045[M-H-Glc] ^-^ [M+Na] ^-^ 803.4558 803.4552 0.75 — BR

[M+Cl] ^-^ 815.4344 815.4354 -1.23 471.3552[M-H-Glc-Fuc] ^-^

[M+HCOO] ^-^ 825.4645 825.4642 0.36

103 29.44 Tenaxin I C_18_H_16_O_7_ [M-H] ^-^ 343.0824 343.0823 0.29 328.0586[M-H-CH_3_] ^-^ [M+H] ^+^ 345.0972 345.0969 0.87 330.0721[M+H-CH_3_] ^+^ SR

313.0333[M-H-2×CH_3_] ^-^ 315.0474[M+H-2×CH_3_] ^+^

104 *^b^* 31.07 Emodin C_15_H_10_O_5_ [M-H] ^-^ 269.0459 269.0455 1.49 241.0502[M-H-CO] ^-^ [M+H] ^+^ 271.0598 271.0601 -1.11 — RRR

225.0560[M-H-CO-O] ^-^

105 32.14 Costunolide C_15_H_20_O_2_ — — — — — [M+H] ^+^ 233.1534 233.1536 -0.86 215.1425[M+H-H_2_O] ^+^ AR

187.1479[M+H-CH_2_O_2_] ^+^

159.1148[M+H-C_3_H_6_O_2_] ^+^

106 32.62 Dehydrocostuslactone C_15_H_18_O_2_ — — — — — [M+H] ^+^ 231.1380 231.1380 0.00 213.1280[M+H-H_2_O] ^+^ AR

185.1329[M+H-CH_2_O_2_] ^+^

157.0987[M+H-C_3_H_6_O_2_] ^+^

107 *^b^* 33.90 Chrysophanol C_15_H_10_O_4_ [M-H] ^-^ 253.0519 253.0506 5.14 — [M+H] ^+^ 255.0657 255.0652 1.96 — RRR

108 *^b^* 35.45 Physcion C_16_H_12_O_5_ — — — — — [M+H] ^+^ 285.0769 285.0757 4.21 — RRR

109 38.92 Ursolic acid C_30_H_48_O_3_ [M-H] ^-^ 455.3522 455.3531 -1.98 — — — — — — GF

110 39.40 Oleanolic acid C_30_H_48_O_3_ [M-H] ^-^ 455.3510 455.3531 -4.61 407.3632[M-H-COOH] ^-^ — — — — — GF

*^a^* RRR, Rhei Radix et Rhizoma; PRA, Paeoniae Radix Alba; GF, Gardeniae Fructus; SR, Scutellariae Radix; BR, Bupleuri Radix; AR, Aucklandiae Radix; CR,

Corydalis Rhizoma;

*^b^* Components identified with reference compounds comparison.

*^c^* Glc, glucose; Rha, rhamnose; Fuc, fucose; GluA, glucuronic acid.
