# Supplementary figures and images for: Qing-Yi Decoction in the Treatment of Acute Pancreatitis: An Integrated Approach Based on Chemical Profile, Network Pharmacology, Molecular Docking and Experimental Evaluation
Source: Front Pharmacol. 2021 Apr 29;12:590994. doi: 10.3389/fphar.2021.590994 (PMC8117095; doi:10.3389/fphar.2021.590994)

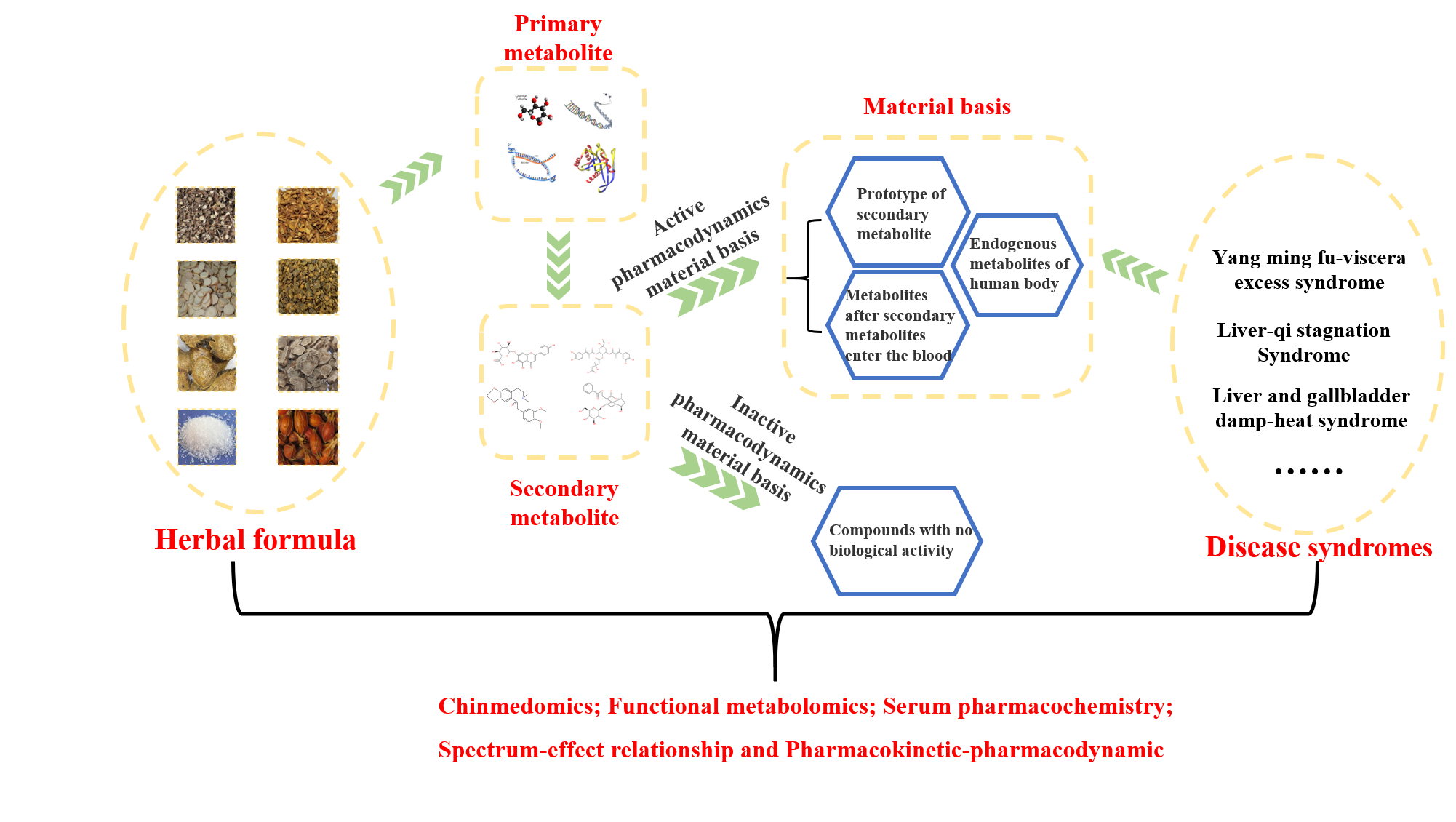

Supplement: Supplementary file 4 [file Image3.TIF]

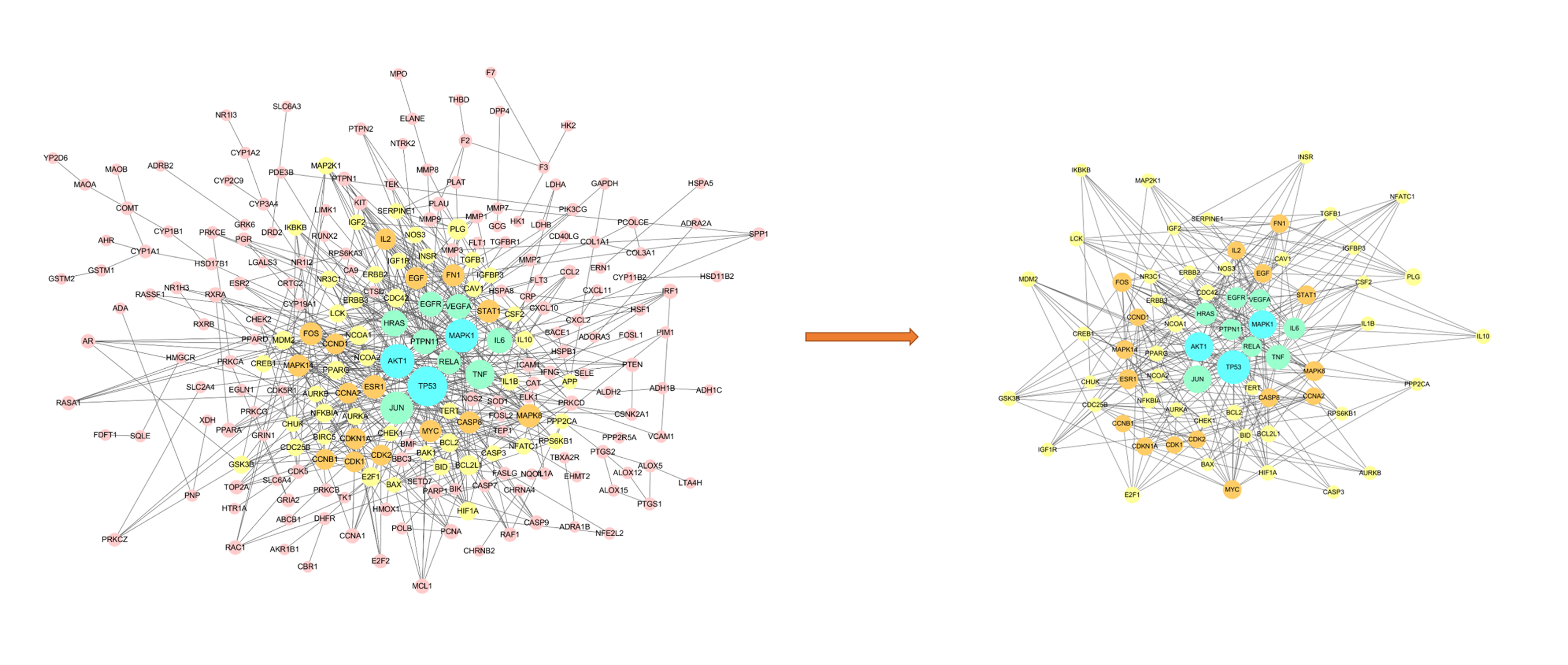

Supplement: Supplementary file 5 [file Image2.TIF]

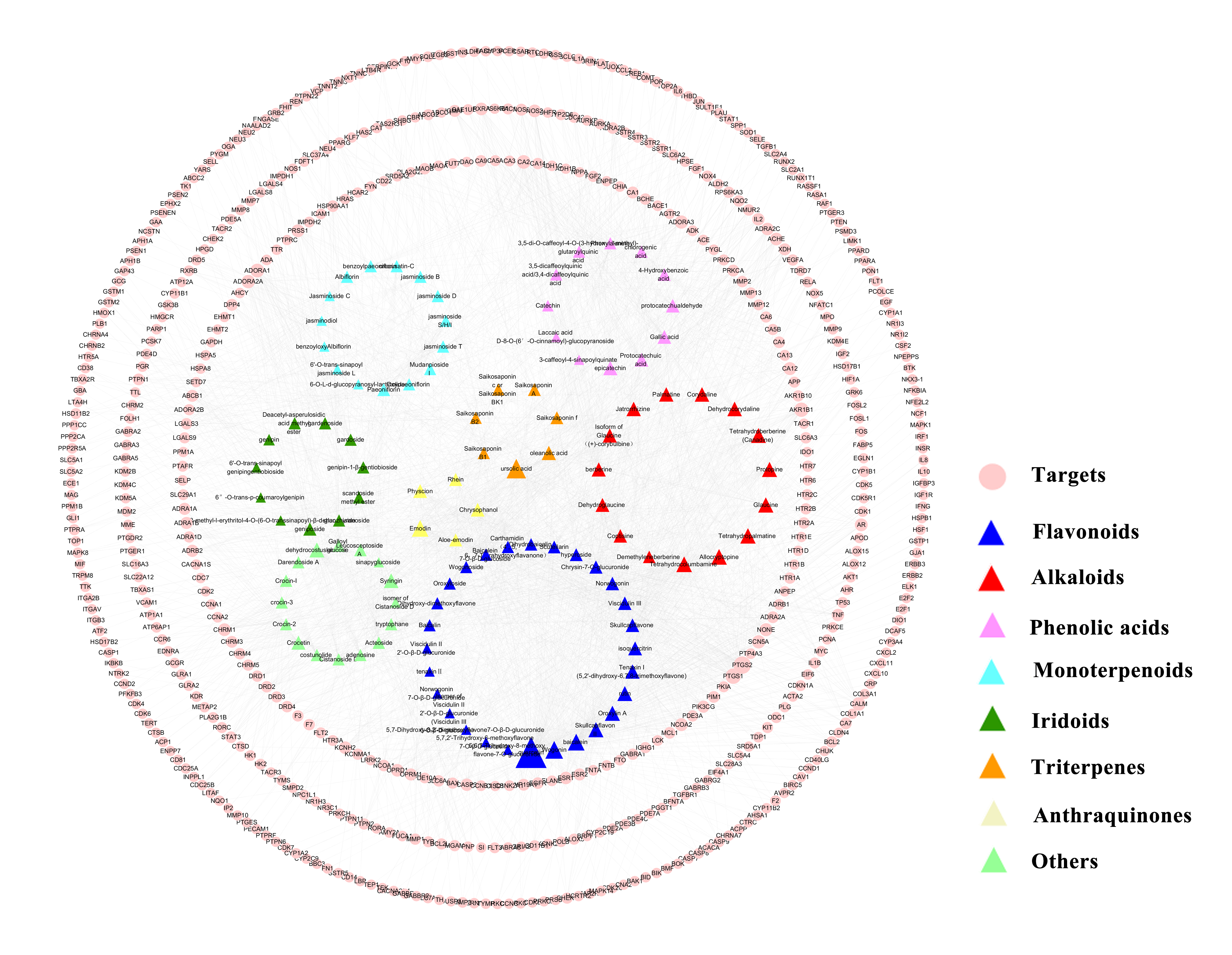

Supplement: Supplementary file 6 [file Image1.TIF]
