# Supplementary material for: Qing-Yi Decoction in the Treatment of Acute Pancreatitis: An Integrated Approach Based on Chemical Profile, Network Pharmacology, Molecular Docking and Experimental Evaluation
Source: Front Pharmacol. 2021 Apr 29;12:590994. doi: 10.3389/fphar.2021.590994 (PMC8117095; doi:10.3389/fphar.2021.590994)
Supplement: Supplementary file 7 [file Table5.docx]

Table 5 PBD ID of each protein

Protein PBD ID Protein PBD ID Protein PBD ID

AKT1（5wby） EGF（1nql） LCK（1lkk）

BAX（5w60） EGFR（5ug9） MAP2K1（3eqc）

BCL2（6gl8） ERBB2（1mfg） MAPK14（2fst）

BCL2L1（3sp7） ERBB3（6op9） MAPK1（6slg）

CASP3（2dko） FN1（2cg7） MYC（6g6k）

CASP8（4jj7） FOS（1a02） NFATC1（5sve）

CCNB1（6gu2） GSK3B（1o6l） NFKBIA（1ikn）

CCND1（2w96） IGF1R（1p4o） RELA（4kv1）

CDC42（4js0） IGF2（3kr3） RPS6KB1（5wbh）

CDK1（6gu2） IKBKB（3brv） SERPINE1（1lj5）

CDKN1A（5e0u） IL10（2ilk） STAT1（3wwt）

CHEK1（1ia8） IL1B（4gai） TGFB1（5vqp）

CHUK（3brt） IL2（4nej） TNF（5uui）

CSF2（6bfs） IL6（1alu） TP53（3d06）

E2F1（6g0p） JUN（5t01） VEGFA（1mkk）
